# Surveying alternative transcripts of disease-associated genes and their cell type specificity – a case study with *ANK3*

**DOI:** 10.1038/s41398-026-04412-9

**Published:** 2026-10-01

**Authors:** Martin Falck, Denis Reis de Assis, Asbjørn Holmgren, Ragnhild Aaløkken, Sarah Åsheim, Hans-Christian Aass, Knut Tomas Dalen, Srdjan Djurovic, Timothy Hughes

**Affiliations:** 1 Department of Medical Genetics, Oslo University Hospital and University of Oslo, Oslo, Norway; 2 FYSCELL - Spatial Immunology and Nanomedicines (Comparative immunology), University of Oslo, Oslo, Norway; 3 Centre for Precision Psychiatry, Division of Mental Health and Addiction, Oslo University Hospital and Institute of Clinical Medicine, University of Oslo, Oslo, Norway; 4 Department of Medical Biochemistry, Oslo University Hospital, Oslo, Norway; 5 Department of Nutrition, Institute of Basic Medical Science, University of Oslo, Oslo, Norway; 6 The Norwegian Transgenic Center, Institute of Basic Medical Science, University of Oslo, Oslo, Norway

**Keywords:** Molecular neuroscience, Bipolar disorder, Clinical genetics

## Abstract

The *ANK3* gene has been conclusively linked to bipolar disorder, but its role in disease etiology remains unclear: both the specific transcript and its expressing cell type remain unclear. This is a near-universal problem for genes associated with polygenic neuropsychiatric disorders. One of the causes of this unfortunate stasis is that human transcript databases have an incomplete list of alternative isoforms of genes and that it is technically challenging to identify the cell-types in which they are expressed. Here, we address this knowledge gap for *ANK3*. *ANK3* is a useful case study because of its rich alternative splicing and important neurobiological role in neurons and oligodendrocytes. We used Oxford nanopore sequencing of full-length transcripts to identify all human and mouse *ANK3* exons. Then, we isolate and sort brain nuclei and use droplet-digital PCR to measure cell-type specific expression of alternatively spliced exons. We confirm that RefSeq transcript annotations of *ANK3* lack many of the alternatively spliced exons and we show that, despite being lowly expressed, these exons are highly evolutionarily conserved and have strong cell type specificity. Elucidating psychiatric disorder etiology will require an overview of the transcripts of all the disorder-associated genes and the cell types in which these transcripts are expressed. This work on *ANK3* illustrates the extent to which such information is currently lacking, ways in which the information can be acquired, and the technical challenges that are encountered in the process.

## Introduction

Genome-wide association studies (GWAS) of polygenic traits produce association signals between polymorphisms and traits at hundreds of genomic loci. The process of fine-mapping is used to predict which gene in the locus drives the genetic association [1]. However, strictly-speaking it is neither the locus nor the fine mapped gene that is associated with the disease. Indeed, most human genes produce multiple transcripts with different transcription start sites (TSS) and alternative splicing of exons, with the expression of these alternative transcripts differing across cell types. Fundamentally, polygenic diseases are caused by complex imbalances in proteins that disrupt specific cellular processes. Thus, it is not the gene that is the driver of the association, but rather the transcripts of the gene that encode these proteins. Thus a comprehensive overview of all the transcripts of an associated gene and the cell types in which they are expressed is a prerequisite for GWAS results to eventually elucidate the etiology of psychiatric disorders [2, 3]. It is tempting to think that single-cell RNAseq can provide such information, but this technology tends to only measure the expression of the most highly expressed genes and does not fully distinguish between alternative transcripts of these genes. In this study, we used the *ANK3* gene (Ankyrin-G protein) as a challenging case study for which we produce such an overview.

**Fig. 1:**
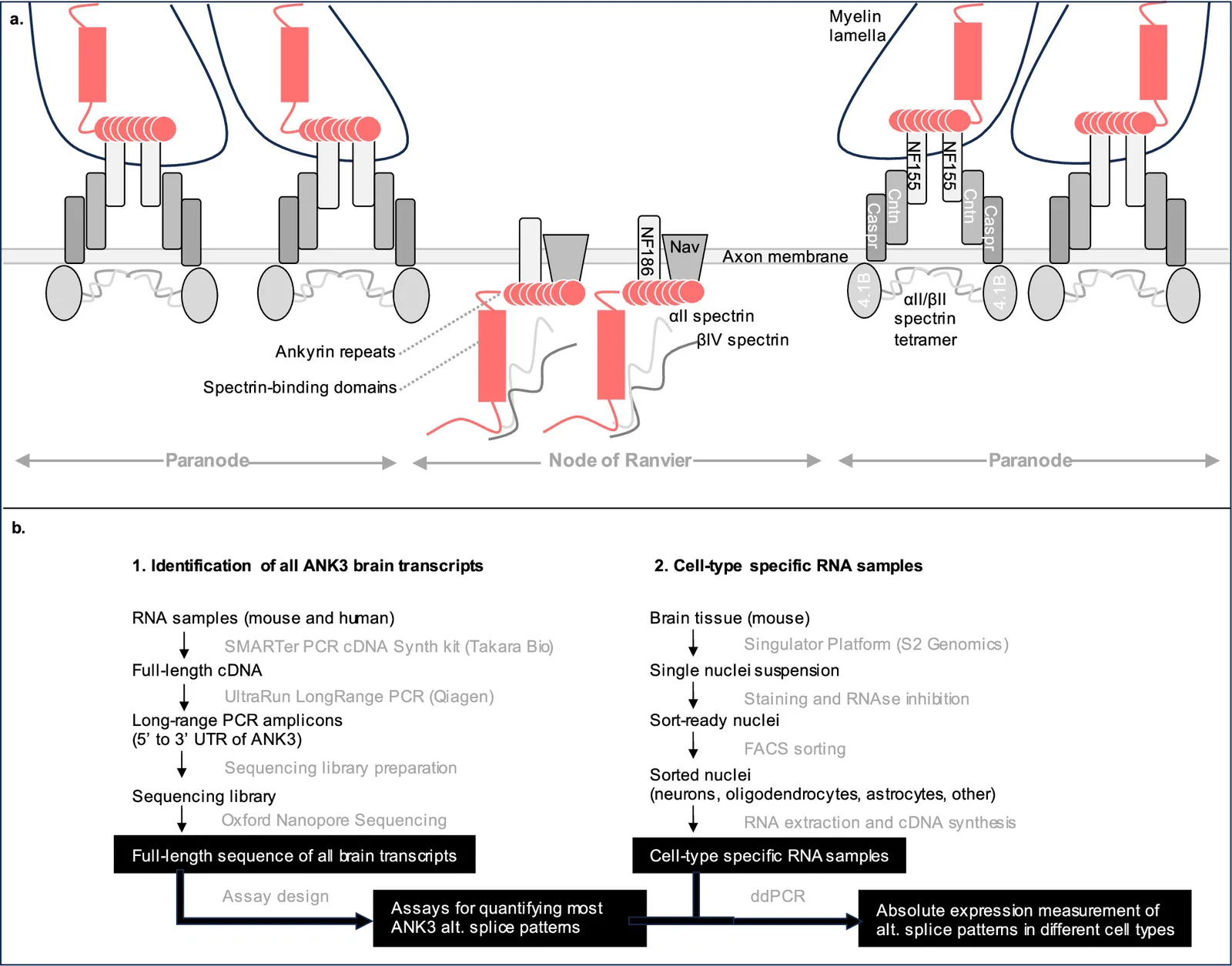
Study overview. **a**. Schematic representation of the axonal membrane and oligodendral lamella at a node de Ranvier and the central role of ANK3 (in red), adapted from [10, 60, 61]. ANK3’s interaction partners at the node and paranode in the central nervous system are: Cntn contactin, Caspr contactin-associated protein, NF neurofascin, Nav voltage-gated sodium channel, 4.1B protein 4.1B. **b**. Overview of the approach to identifying and quantifying all *ANK3* transcripts and their expression in the main brain cell types.

Ankyrin-G is a scaffolding adapter protein that links membrane proteins to the β-spectrin/actin cytoskeleton [4, 5]. The Ankyrin-G protein exists in many alternative isoforms expressed in different cell types [6]. In neurons, its most prominent role is as a master organizer of the axon initial segment and nodes of Ranvier. Here, the giant *ANK3* isoforms (270/480 kDa) mediate the correct clustering of sodium channels, which is essential for the initiation of the action potential and its propagation [7]. *ANK3* also modulates dendritic spine structure and function, probably as a perisynaptic scaffold and barrier within the spine neck [8, 9]. In oligodendrocytes (Fig. 1a), *ANK3* is essential to the assembly and maintenance of the paranodal axoglial junction [10, 11], whilst in astrocytes, the protein is known to be expressed, but its function remains unclear.

Large well-powered GWAS have consistently associated common SNP-variation in *ANK3* with severe polygenic neuropsychiatric disease, most notably bipolar disorder (BD) [12–16] and low frequency variants with functional effects on specific isoforms have been associated with the disorder. For instance, a specific effect of a BD-associated haplotype on *ANK3* isoform expression has been detected in human brain [17]. Further, we have identified a rare loss-of-function splice-site SNP (rs41283526*G) in a minor isoform of *ANK3* (incorporating exon ENSE00001786716) as protective of BD [18] and we have shown that risk alleles identified by BD GWAS are located near the transcription start site of this isoform and are significantly associated with its elevated expression [19]. There is also an indication of a link with schizophrenia, but the evidence is less compelling [20–22], and reports of patients suffering from neurodevelopmental disorders such as attention deficit hyperactivity disorder, autism spectrum disorder, and intellectual disability, where deleterious mutations in the *ANK3* gene are likely causal: compound heterozygous [23], de novo [24, 25], heterozygous [26, 27], and homozygous [28, 29]. Finally, there is evidence of a significant enrichment of de novo potentially disease-causing variants in the proteins interacting with Ankyrin-G, notably subunits of sodium and potassium channels [30].

Mouse models have been extensively used to study the knock-out (or knock-down) of the whole *ANK3* gene (or of specific isoforms) [23]. Effects are typically significant, ranging from changes in cellular structures and animal behavior for certain cell- or isoform-specific models [8, 10, 11, 31] to post-natal lethality for loss of the whole gene [32]. Interestingly, lithium, which is the treatment of choice for BD [33, 34], has been shown to modulate the function of ANK3 [35] and rescue some aspects of ANK3 deficiency in mouse models [36–38] and a SNP in *ANK3* is a candidate genetic biomarker for the efficiency of Liafensine in treatment-resistant depression [39].

In summary, ANK3 is essential to cellular structures in dendritic spines, axon initial segments and nodes of Ranvier which are integral to the electrophysiological properties of neurons. Further, variation in the gene has been robustly associated with mono- and polygenic neuropsychiatric disease. These characteristics have led to *ANK3* being one of the best-studied bipolar susceptibility genes, with mouse studies distinguishing between the isoforms with major size differences in neurons (190/270/480 kDa). However, *ANK3* has many smaller alternatively spliced exons which are poorly characterized. It is not necessarily the largest transcripts of a gene, or those that are most highly expressed, that are causal for pathology, so there is a risk that *ANK3* isoforms relevant to disease have been overlooked. To remedy this, we combined Oxford Nanopore sequencing of full-length cDNA, FACS sorting of brain cell types, and droplet digital PCR (ddPCR) to: 1) obtain a complete overview of the isoforms expressed in the brain (irrespective of length or expression level), 2) determine the extent to which mouse and human express the same isoforms, 3) determine the expression levels of the most common splice patterns in different mouse brain cell types (Fig. 1b). This overview of *ANK3* transcripts provides a basis for a more complete understanding of *ANK3*’s role in normal human neurobiology and its association with disease.

## Methods

### Sequencing of full-length human and mouse *ANK3* transcripts

#### cDNA and long-range amplification

**Fig. 2:**
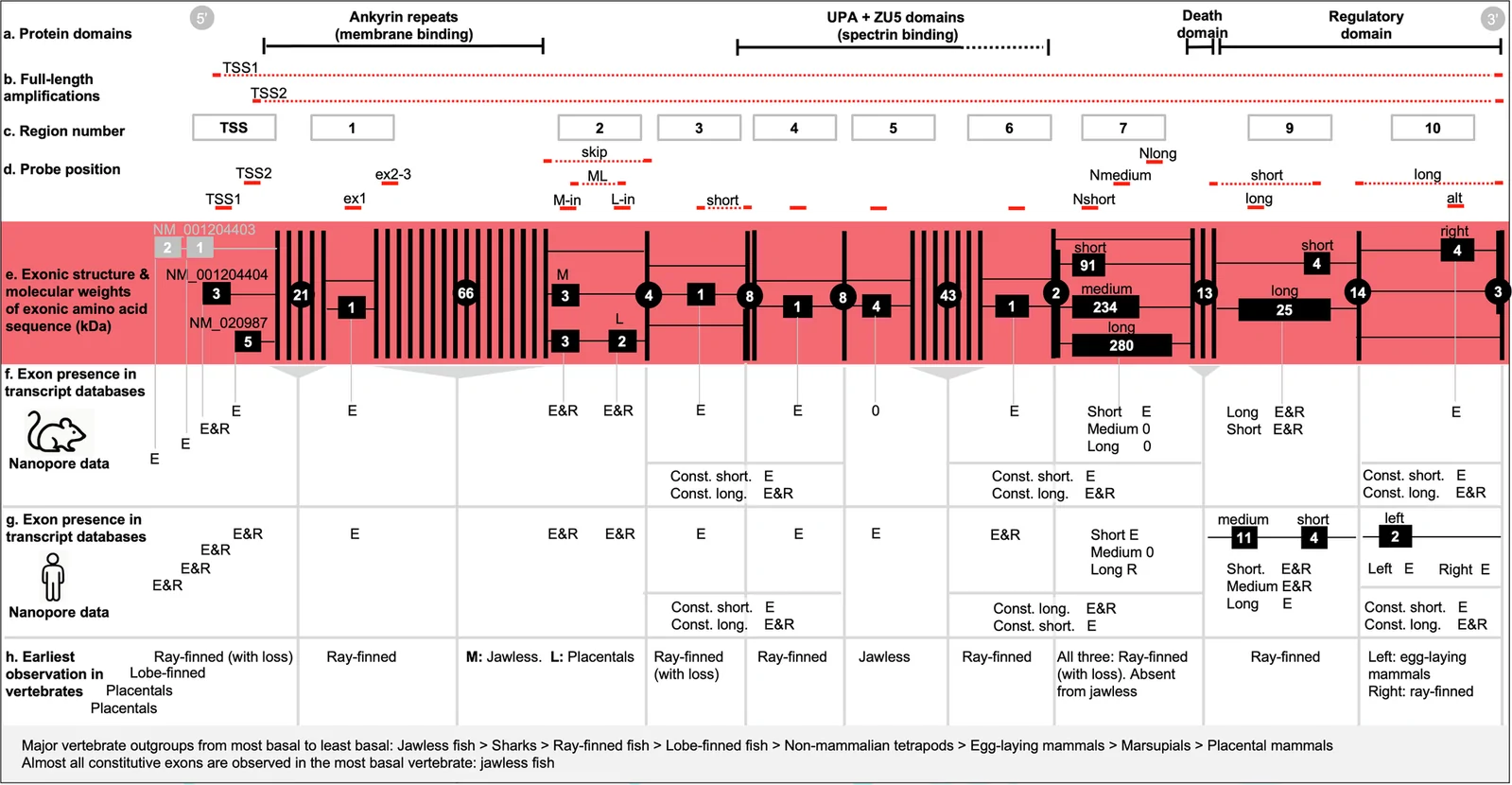
Full-length transcript Nanopore sequencing and ddPCR probes. **a**. Protein domains: as annotated in Ensembl with additions from [5]. **b**. Full-length amplifications: primer positions. **c**. Region number: numbering of alternatively spliced regions. Region 8 is deliberately absent (it was putative, but not confirmed). In each region, skipping the alternatively spliced exon is usually the most highly expressed splice pattern. **d**. Expression probe position: schematic of ddPCR probe positions. **e**. Exonic structure and molecular weights: black vertical bars depict constitutive exons. Numbers displayed in alternatively spliced exons or in circles over constitutive exons are the molecular weights of the encoded peptide and are broadly proportional to the amino acid length. **f**. Status of mouse and **g**. human exons observed in nanopore data in transcript databases: Present in Ensembl (E), RefSeq (R), Ensembl and RefSeq (E&R), neither (0). There are 3 constitutive (const.) exons with both a short and a longer form between regions 3 and 4, 6 and 7, and at the end of region 10 (which are 3, 15 and 36 nucleotides longer). The figure only includes protein coding exons (exons that consist of 100% untranslated region are not represented). **h**. Earliest observation in vertebrates: name of most basal vertebrate outgroup in which exon sequence is conserved, inferred from comparative genomics track from the UCSC Genome: 100 vertebrates genomes aligned with MultiZ [62]. See supplementary materials for details.

We obtained RNA from human corpus callosum and frontal lobe (TakaraBio, #636133 and #636165). The same brain regions were dissected from mice and RNA was isolated using the RNeasy Plus Mini Kit (QIAGEN #74136). cDNA was synthesized using 1 µg total RNA, per brain region, using the SMARTer® PCR cDNA Synthesis Kit (TakaraBio). 10 cycles of general amplification were performed using the UltraRun LongRange PCR Kit (QIAGEN) according to manufacturer’s instructions, but with an extension phase length of 13 min to allow amplification of particularly large *ANK3* products. *ANK3*-specific amplification was performed with 28 × 13 min long extension cycles using a time increase per subsequent cycle (+20 s) using primers designed for either transcription start site 1 (TSS1) or TSS2 together with a common reverse primer (Fig. 2b and Table S1). We analyzed products on the 2100 Bioanalyzer system utilizing the Agilent DNA 12000 kit (Agilent Technologies) (Fig. S1). Products were then cleaned with Agencourt AMPure XP beads and the below 3 kb fragment fraction was reduced by using a 40:60 bead:sample ratio (Beckman Coulter).

*Nanopore MinION:* For human samples, the sample preparation process was performed with the SQK-LSK109 ligation sequencing kit and multiplexed with the EXP-NBD196 native barcoding kit (Oxford Nanopore). The R9.4.1 flow cell was primed with the EXP-FLP002 flow cell priming kit from Nanopore, and the multiplexed samples were run on the MinION Mk1C machine in fast mode for 72 h. Fast5 files obtained from the MinION were basecalled with high-accuracy (HAC) using GPU-Guppy (Nanopore) running in Ubuntu 20.04 and Python 3.7.12. We filtered the resulting Fastq files using seqkit [40] (2.3.1): we only retained sequences that contained both forward and reverse primers (*ANK3* amplification primers for either TSS1 or TSS2 with the reverse primer, allowing for one nucleotide mismatch) and were at least 3000 nucleotides in length (Table S2). The sample files were aligned to the GRCh38 reference genome using minimap2 [41] (2.24) in long read mRNA mode (-ax splice) with the maximum intron size (-G) set to 600k. We used samtools [42] to sort the alignments and remove any non-primary alignments. The mouse samples were run on the updated flow cell platform R10.4.1 and downstream analysis was performed in an analogous way.

*ANK3 isoform analysis:* We took two different approaches to analyzing the four sequencing datasets: a manual inspection of alternatively spliced regions in IGV and a computational analysis of all isoforms. In the manual analysis, we loaded in turn each of the four datasets of sequenced transcripts into IGV [43] and identified by inspection all regions affected by alternative splicing of exons and their read coverage (Supplementary Tables S3, S4). We then used the Ensembl [44] and RefSeq [45] databases to annotate the exons within these alternatively spliced regions (Fig. 2f, g). In the computational analysis of all isoforms, the goal was to build a catalogue of all the different ways in which these alternatively spliced regions combine into different isoforms. In brief, we used FLAIR [46], which was specifically developed for use with long-read technologies, to computationally define and analyze the *ANK3* isoforms in each sample. However, we needed to make a custom adjustment to the reference GTF files used by the tool in order to correctly handle idiosyncrasies of *ANK3* such as the very long neuronal exon and the stretch of 16 constitutive exons encoding the ankyrin repeats (see supplementary materials).

### Nuclei isolation and flow cytometric analysis and sorting

#### Animals

Experiments were conducted using WT C57BL/6 mice (Jackson Laboratories). Adult mice (from 2 to 4 months-old) of both sexes were housed in cages with a 12 h light-dark cycle and *ad libitum* access to water and food. Mice were killed by cervical dislocation and extracted brains were transferred to 5 mL Eppendorf tubes, and kept at −80 °C.

#### Brain dissection and nuclei isolation

Mouse brains were thawed and cortical tissue was dissected for nuclei isolation using a Singulator 100 System and Singulator Nuclei Isolation Kit (S2 Genomics, #100-060-817). 50–100 mg of cortical tissue, 0.2 U/mL ribonuclease inhibitor (RI, Invitrogen, #10777-019), 0.04% Triton X 100, and 5 µl of protease/phosphatase inhibitors were added into the cartridge chamber, and nuclei were isolated according to the manufacturer’s protocol. The isolated nuclei were kept in nuclei storage reagent (NSR, S2 Genomics, #100-063-405 or #100-063-623) supplemented with 0.2 U/mL RI, inside 5 mL Eppendorf tubes on ice. Nuclei were counted using NucleoCassette (ChemoMetec, #941-0001) fitted into a NucleoCounter NC-200 (ChemoMetec). RNA and DNA content of isolated nuclei were identified by adding SYTO9 (Green Fluorescent Nucleic Acid Stain, Invitrogen, #S34854) and DAPI (ThermoFisher, #62248) (Fig. S8)

#### Immunostaining and flow cytometric sorting

Freshly isolated nuclei were centrifuged at 500 x *g* for 5 min at 4 °C and resuspended in NSR, and 700,000 nuclei were transferred to 1.5 mL Eppendorf® Protein LoBind tubes (Eppendorf, #0030108116) in 50 μl ice-cold dPBS (no Ca^2+^, no Mg^2+^, Sigma-Aldrich, #14190144) with 0.1% Triton X-100. Nuclei were stained with 1:100 4′,6-diamidino-2-phenylindole (DAPI) 1 mg/mL (Thermo Scientific, #62248), anti-NeuN-AF488 (neurons), anti-Olig2-AF647 (oligodendrocytes), anti-SOX9-PE (astrocytes) (Table S5). The tubes were incubated on ice for 45 min sheltered from light. Stained nuclei were centrifuged at 500 x *g* for 5 min at 4 °C, the supernatants discarded, and nuclei pellets were resuspended in 150 µl dPBS prior to flow cytometric analysis and sorting. Sample acquisition and cell sorting were conducted on a FACSMelody™ cell sorter powered with the Chorus software (BD Biosciences). See details in Supplementary Materials. After sorting, nuclei from each different cell type were snap frozen on dry ice and lysed with RLT plus buffer supplemented with 10% β-mercaptoethanol. A total of four FACS experiments were needed to provide sufficient quantities of RNA for the ddPCR experiments.

#### RNA extraction

RNA was extracted from lysed nuclei using RNeasy Plus Micro kit (Qiagen, #74004), and total RNA was eluted in RNAse-free water. RNA concentrations were measured by a Nanodrop 1000 Spectrophotometer V3.8 (Thermo Fisher Scientific) and RNA integrity numbers (RIN) were measured by a Bioanalyzer 2100 using a RNA Pico chip (Agilent Technologies, #5067-1513).

### Expression by droplet digital PCR

#### cDNA synthesis and ddPCR

cDNA synthesis was performed on 4.4 ng of each sample with the High-Capacity cDNA Reverse Transcription Kit (Applied Biosystems, #4368814), using 20 μL reaction volume. Assays for *Gfap*, *Sox9*, *Top1* and *Ank3* isoforms consisting of forward primer, reverse primer and probe labelled with FAM or HEX/VIC were mixed and concentrations were adjusted to allow for amplitude-based multiplexing of 3–4 assays in each well (Fig. 2d and Table S8). A total of 8 FACS experiments from separate mouse individuals were used. Since the astrocyte RNA material was scarce, each *Ank3* isoform was only measured in 4 astrocyte replicates. The ddPCR reactions were prepared with primers, probes, 1× ddPCR Supermix for Probes No dUTP (Bio-Rad) and cDNA from 1 ng RNA. Droplets were generated on the QX200 AutoDG Droplet Generator (Bio-Rad), then transferred to a thermal cycler, and subjected to the following PCR profile suggested by the manufacturer: an initial melting step at 95 °C for 10 min; then 40 cycles of 94 °C for 30 s, 60 °C for 60 s, then 98 °C for 10 min and a 4 °C infinity hold. Droplet fluorescence was measured on the QX200 Droplet Reader and analyzed with the QX manager Software Standard Edition v2.1 (Bio-Rad).

#### Reference gene expression stability in mouse cortex

We performed an analysis of reference gene expression stability in order to select a gene with a stable expression and a similar expression level to *Ank3*. We obtained RNAseq read counts from an in-house dataset of adult mouse frontal cortex (*n* = 16), as well as published read counts of the same tissue (*n* = 86) [47]. Reads mapped to genes were between-sample normalized using the weighted trimmed mean of M-values (TMM) with edgeR [48]. Genes with low expression (>10 counts in >30% of the samples, converted to CPM for the median library size) were filtered out with the “filterByExpr”- function in the edgeR package. Gene counts were converted to log2 counts per million (CPM) values with the cpm function in the edgeR package. Reference gene candidates (*n* = 15) were obtained from Rydbirk et al. [49] and previous in-house experiments [50]. For each dataset, we calculated the coefficient of variation (CV) [51], sd(cpm)/mean(cpm), on the log2 scale.

#### Top1 reference gene adjustment and normalization

Reference gene expression stability was analyzed a priori on bulk frontal cortex, and the reference gene *Top1* was chosen, but we found the *Top1* expression to be dependent on cell type. In order to make comparisons of expression levels across cell types, we used the median *Top1* expression within each cell type to calculate a factor with which each sample’s *Top1* was normalized (Fig. S11). Expression levels of all targets were converted to log2 (copies per ng RNA) and normalized with the cell type-adjusted level of *Top1*.

*Astrocyte enrichment and Ank3 isoform expression between cell types:* Astrocyte enrichment was assessed by comparing the expression of the astrocyte marker genes *Sox9* and *Gfap* in astrocytes (SOX9^+^ nuclei) and unsorted (DAPI) from each nuclei sort experiment, with a paired t-test. The “limma” package [52] was used to test for differentially expressed *Ank3* isoforms between neurons, oligodendrocytes and astrocytes. For each contrast, empirical Bayes moderated t-statistics were calculated (Table S11), and their *p*-values were adjusted for multiple testing, with FDR < 0.01.

## Results

### Sequencing of full-length human and mouse *ANK3* transcripts

There are 10 different regions of the *ANK3* gene in which alternatively spliced exons are found (Figs. 2c and e, S2). In most of these regions the alternatively spliced exon is either included or not (skipped), e.g. regions 1, 4 and 5, whilst other regions have more complex splice patterns e.g. regions 2 or 7 (neuronal exon). These different alternative splice patterns in each region combine to form a complete transcript which is one of many different isoforms of the *ANK3* gene.

In both human and mouse, most of these alternatively spliced exons are predicted in the Ensembl annotation, but have lacked the biological evidence to be entered into RefSeq. Our Oxford Nanopore sequencing of the amplified full-length transcripts of *ANK3* provides the evidence that these are true exons (Figs. 2f and g., S2 and S3). For example, we confirm that TSS2 is incorporated into mouse *Ank3* transcripts despite currently not being in any RefSeq transcripts.

We also note that there are 3 constitutive exons with both a short and a longer form between regions 3/4, 6/7, and at the end of region 10 (which are 3, 15 and 36 nucleotides longer). These different exon lengths are also predicted by Ensembl, but the short forms are not included in RefSeq (Figs. 2f and g, S2 and S3) despite being observed in a substantial fraction of our transcripts reads (in both human and mouse).

This large number of alternative splice regions can theoretically combine into thousands of possible isoforms. In our data, we find that the vast majority of transcripts are one of 26 isoforms and that among these there is one that is clearly dominant, approximately 5–10 that are moderately expressed, and the rest that have low expression (Figs. S3–6). Few of these isoforms incorporate the neuronal exon, but this could be explained by a bias against PCR amplification of the long transcript that results from the incorporation of this very large exon. RefSeq only records seven different isoforms of *Ank3* in mouse and five in human which indicates that this database has only annotated a small fraction of the true isoforms of *Ank3*.

In each splice region, the most common pattern is the one that skips the alternatively spliced exon, consequently the Nanopore data suggests that the alternatively spliced exons are lowly expressed (Fig. 2e and Table S3–4).

There is almost perfect concordance in the splice patterns of the different regions between human and mouse (Fig. 2f and g) which indicates that there is high evolutionary conservation of isoforms and that mouse should be a good model for studying the neurobiology of human *ANK3*. Further, our comparative genomics analysis suggests that these exons are well-conserved throughout vertebrates (Fig. 2h) suggesting that they encode essential functions, but that this functionality is only sporadically required in *ANK3* transcripts.

### Nuclei isolation and flow cytometric sorting

**Fig. 3:**
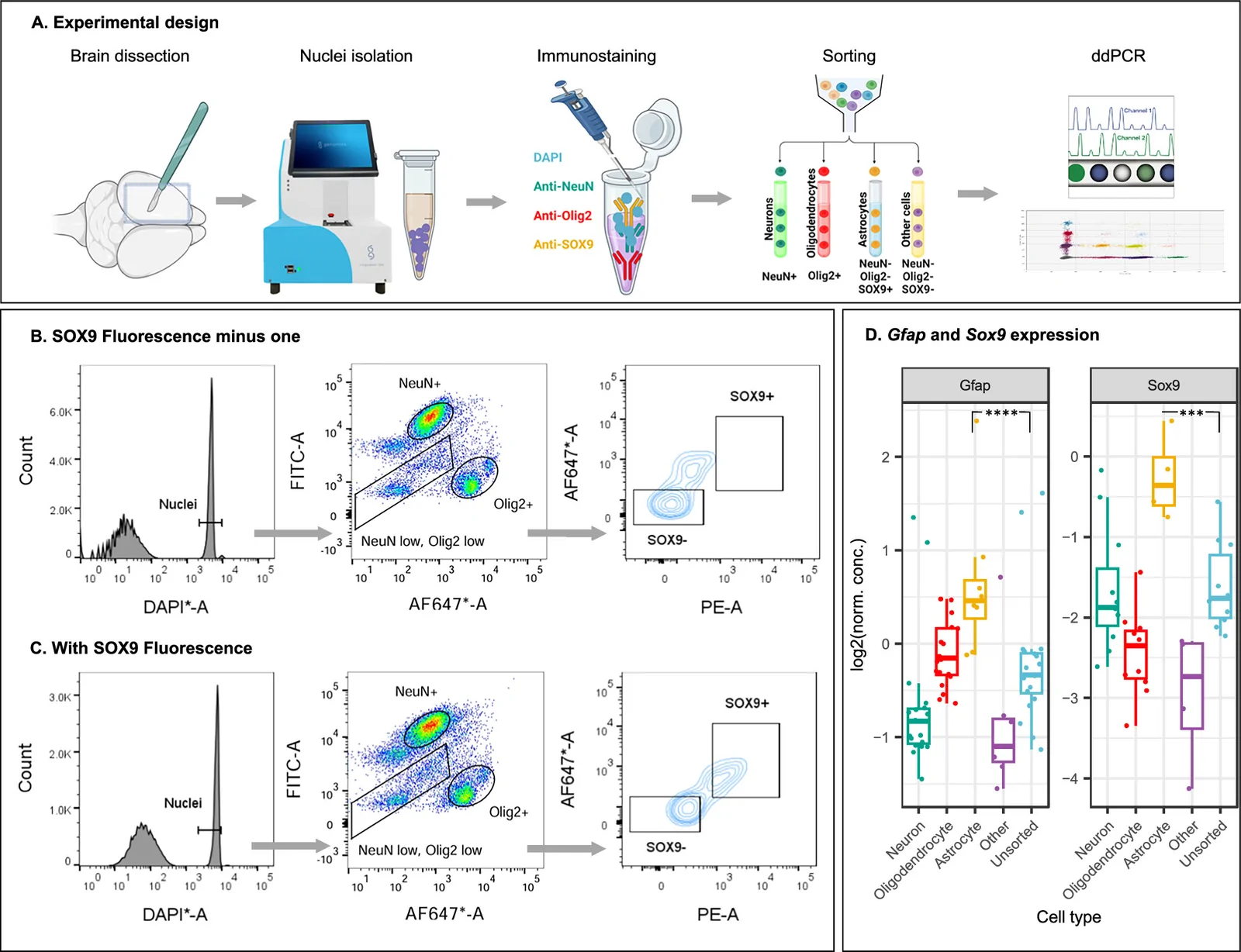
Nuclei sorting from mouse brain cortex. **A**. Scheme showing the experiment design, including brain cortex dissection, nuclei isolation, immunostaining of nuclei with the specific cellular type antibodies against NeuN, Olig2 and SOX9, sorting of nuclei from different cell populations, and ddPCR to identify the expression of *ANK3* isoforms in nuclei from each cell population. Elements of this panel were created in BioRender. **B**. Gates from nuclei stained with antibodies anti-NeuN and anti-Olig2, but without anti-SOX9 (SOX9 fluorescence minus one, FMO tube) as a negative control for SOX9 expression. **c**. Gates obtained from nuclei stained with antibodies anti-NeuN, anti-Olig2, and anti-SOX9. First, nuclei singlets were identified based on DAPI staining, then three gates were plotted: NeuN+ (neurons), Olig2+ (oligodendrocytes), and NeuN low, Olig2 low. Finally, the gates SOX9+ (astrocytes) and SOX9- (other cells) were derived from the gate NeuN low, Olig2 low, and the different subpopulations of nuclei were sorted. **D**. Astrocyte markers *Gfap* and *Sox9* RNA expression in the sorted nuclei populations, in log2 of the normalized concentration (copies per μl normalized to the reference gene *Top1*, adjusted for cell type). Statistical significance tested between the astrocyte population and unsorted nuclei (mixture of all nuclei), ***: *p* < 0.001, ****: *p* < 0.0001.

The main neural cell types of adult mice possess long processes that are tightly interwoven, making it impossible to obtain a suspension of intact single cells. Instead, we dissected cortical tissue, isolated nuclei, and performed immunostaining, acquisition, and nuclei sorting (Fig. 3A).

Isolated nuclei were stained to visualize RNA and DNA content, confirming the integrity and molecular content of the isolated nuclei (Fig. S8). Immediately after immunostaining, the nuclei were further analyzed by flow cytometry (Fig. 3B and C). Single nuclei were identified in a pulse area histogram of DAPI-stained nuclei. Subpopulations derived from neurons and oligodendrocytes were identified in a density plot of pulse area of anti-NeuN-AF488 versus pulse area of anti-Olig2-AF647. Furthermore, nuclei derived from astrocytes were identified (NeuN-low and Olig2-low) as SOX9-positive. A fluorescence-minus-one (FMO) control was used to ensure correct identification of the true SOX9-positive astrocytes (Fig. 3B).

Four nuclei populations were sorted simultaneously: those derived from neurons (NeuN-positive), oligodendrocytes (Olig2-positive), astrocytes (Olig2-low, NeuN-low, and SOX9-positive), and a population of SOX9-negative nuclei (Olig2 low, NeuN low) (Fig. 3C). Additionally, the entire single nuclei population (DAPI-stained) was also sorted as a control sample for cortical tissue.

Re-analysis of sorted cells confirmed high purity, with both neurons and oligodendrocytes exhibiting a purity of above 95% (Fig. S9). Due to the low number of cells (Table S6), a purity test was not performed on the sorted astrocyte population. However, we confirmed high purity of the astrocytes (Olig2-low, NeuN-low, and SOX9-positive) with the GFAP RNA enrichment performed using ddPCR: both markers were significantly enriched in astrocytes relative to oligodendrocytes and neurons, but more importantly, relative to the unsorted fraction (GFAP 3.3-fold, p = 1.0E-5; SOX9 5.0-fold, p = 7.0E-4) (Fig. 3D, Table S11).

### Expression by droplet digital PCR

The Nanopore data confirmed the presence of 10 alternatively spliced regions and it also provided an indication of the low expression of most of these exons (Figs. 2 and S2). However, the accuracy of these expression measures is limited due to: a bias against the successful amplification of very long transcripts and the absence of a correction for the quality and quantity of the input DNA. We overcome these limitations by synthesizing cDNA in the standard way (not full-length and no amplification), use a reference gene to normalize the expression values, and measure the expression by ddPCR which is a gold standard for accurate measurement of molecules present at low concentrations [53].

The RNA extracted from each subpopulation of nuclei (NeuN+, Olig2+, SOX9+, and SOX9-) all had an RNA integrity number (RIN) higher than 7.0 (Table S7). We used publicly available datasets to identify a good endogenous control gene: stable in expression and an expression level similar to *Ank3*. Reference genes that are often used, such as *Gapdh*, show a very high expression level relative to *Ank3* and considerable inter-individual variation. We identified *Top1* as providing a good compromise between an appropriate expression level and low variation (Fig. S10, Tables S9–10).

#### Within cell type comparisons

**Fig. 4:**
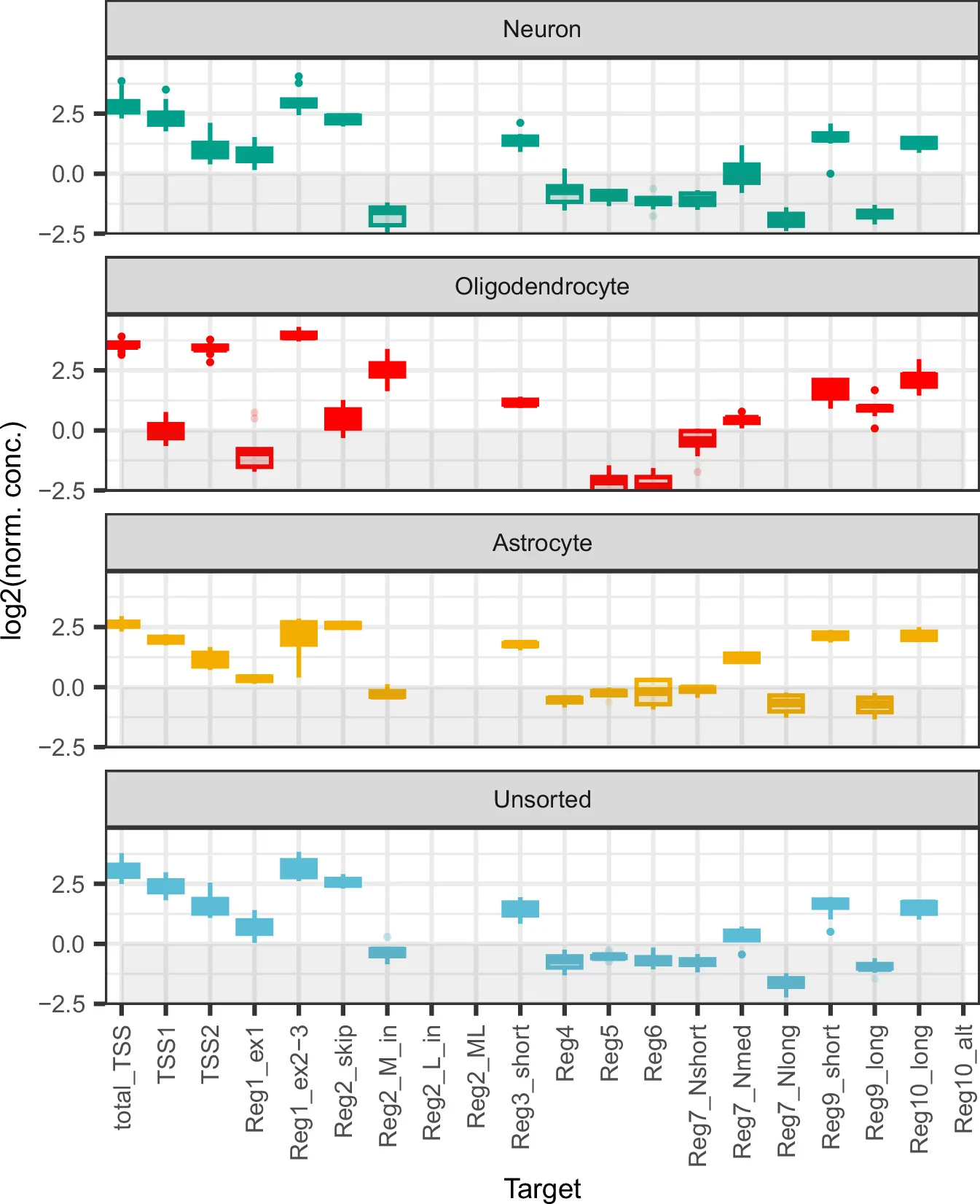
Expression of *Ank3* isoforms within cell populations. Expression of the measured *Ank3* isoforms for each cell type, in log2 counts per ng RNA normalised to the reference gene *Top1* (not adjusted by cell type). Grey boxes indicate log2(norm.conc) <0, to highlight the isoforms that are highest expressed for each cell type. Values < −2.5 are not displayed.

We have separate expression measures for TSS1 and TSS2, and note that the total expression (total_TSS = sum of TSS1 and TSS2) is approximately equal to the constitutive probe (reg1_ex2-3), confirming that these are the two main TSSes in neural cell types (Fig. 4). All three cell types express *Ank3* at a similar level, with the dominant TSS being TSS1 in neurons and astrocytes, and TSS2 in oligodendrocytes. The only alternatively spliced exon with a high expression is reg2_M_in, all other alternatively spliced exons are expressed in a small minority of transcripts or not expressed at all. All of the different forms of the neuronal exon are detected, but it was surprising to observe that they were not exclusive to neurons and even in neurons they are only incorporated into a small fraction of transcripts (Fig. 4).

#### Between cell type comparisons

**Fig. 5:**
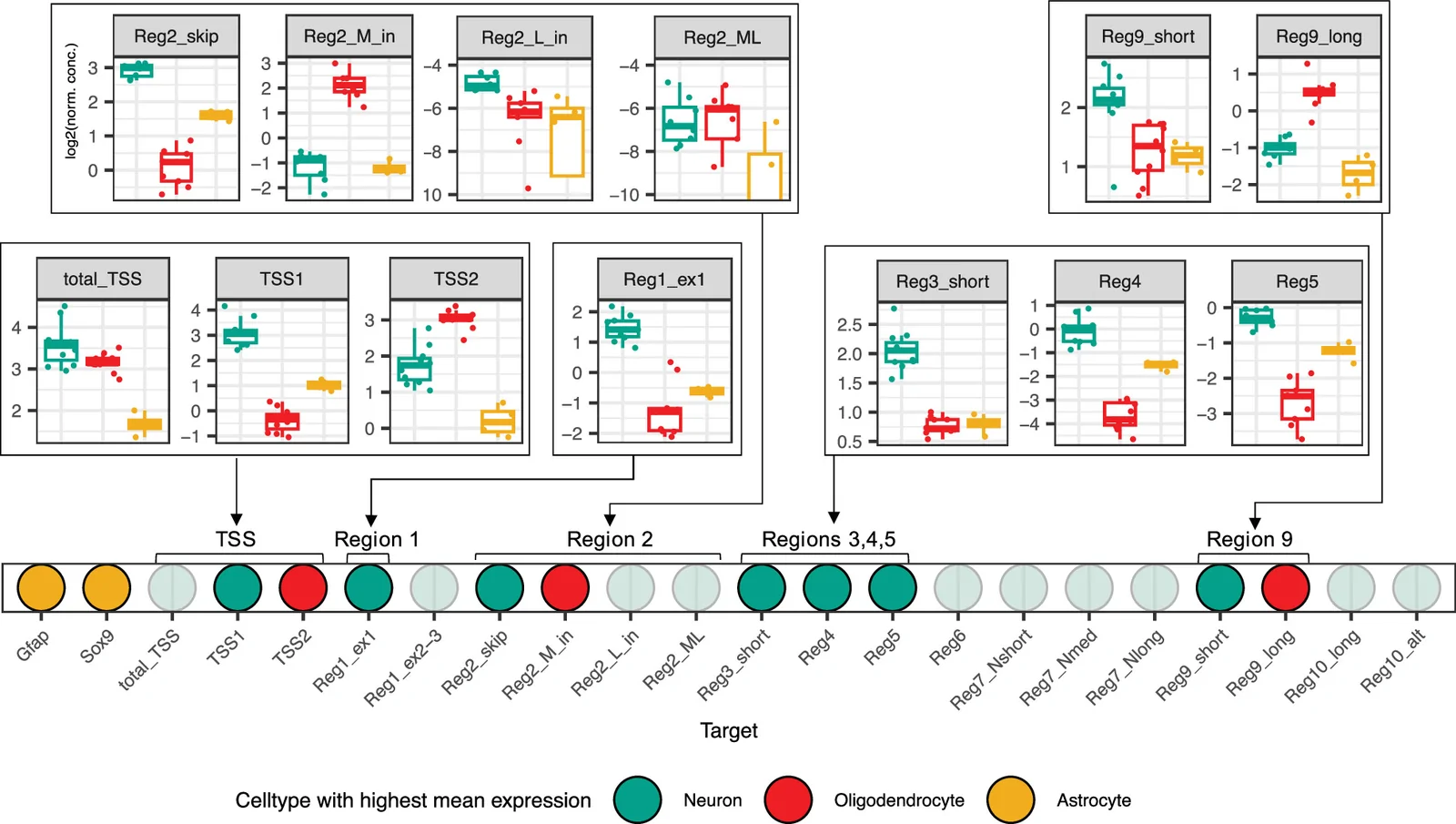
Expression of *Ank3* isoforms between cell populations. Comparison of the expression of several *Ank3* isoforms in different cell type populations. Expression in log2 counts per ng RNA, normalized to the adjusted *Top1* reference gene. The data represents 8 biological replicates. Because of RNA scarcity, each *Ank3* isoform was only measured in 4 astrocyte replicates. Isoforms with significant differences between cell types are highlighted with circles below. Color in circles denotes the cell type with highest expression. Solid circles indicate significant differences to the cell population with second highest expression (FDR < 0.01) whilst transparent circles indicate non-significant differences. Plots for all probes are available in Fig. S11.

We also wanted to perform between cell type comparisons of expression for a given probe. However, we found that the expression of *Top1* differed between cell types (systematically across independent sortings, Fig. S11a). Therefore, we calculated adjustment factors to compensate for this (Fig. S11b, Table S12) and applied them to the normalized expression measurements. The resulting levels confirm two overarching features of *Ank3* expression (supplementary Fig. S11c): 1) TSS1 + TSS2 capture all isoforms as the expression level is in line with the constitutive probe; 2) total *Ank3* expression is on average higher in oligodendrocytes than in neurons. Further, the adjusted expression values suggest that seven alternatively-spliced exons have expression levels that are statistically significantly many times higher in some cell types (Fig. 5 and details in Fig. S12 and Table S13): TSSes, reg1, reg2, reg3, reg4, reg5. One cannot claim perfect cell type specificity, but it comes quite close. In region 9, we observe a preferential use of the short or long exon in two different cell types.

## Discussion

We aimed to produce an overview of the alternatively spliced exons of *ANK3* and their expression levels in the main neural cell types and, in the process, to identify the technical challenges that arise in obtaining this information. Our main biological findings are three-fold.

First, many of the alternatively spliced exons, which are predicted by Ensembl but absent from RefSeq, are confirmed by our Nanopore sequencing and we estimate that they combine into at least 26 different isoforms of *ANK3*, most with low to medium expression, but with one dominant isoform from each TSS (Figs. S4–7).

Second, there is very high concordance between the mouse and the human isoforms. Most research on ANK3 function is performed in mice with the assumption that findings transfer to human neurobiology. In mice, ANK3 has been shown to play a central role in essential neurobiological properties. In neurons, ANK3 modulates the structure and function of dendritic spines [8, 9] and acts as a master organizer of the axon initial segment and nodes of Ranvier where it mediates the correct clustering of sodium channels which is essential for the initiation of the action potential and its propagation [7]. In oligodendrocytes, ANK3 is essential to the assembly and maintenance of the paranodal axoglial junction [10, 11]. Our demonstration of the mouse-human concordance in isoforms indicates that these findings are most likely also true in human and that the mouse is a good model for any future work on the alternatively spliced exons of *ANK3*.

Ideally, it would have been possible to accurately measure the expression of each isoform. However, the nanopore data is not suitable for this purpose. Instead, we used ddPCR to quantify the expression of alternatively spliced exons (irrespective of the isoform in which they were incorporated). Our third result is that the alternatively spliced exons are mostly expressed at low levels (with the exception of region 2) but their sequences are highly evolutionarily conserved and about half of them have a strong degree of cell type specificity, which suggest an important cell-type specific functional role. In all likelihood, many of these short exons harbor eukaryotic linear motifs which are sites for functional modifications such as cleavage, phosphorylation and other post-translational modifications [54] which may be involved in ANK3’s association with psychiatric disease.

Our analysis focused on the protein-coding exon structure of *ANK3*. However, alternative usage of 5′ and 3′ untranslated regions could introduce additional layers of post-transcriptional regulation. Future work examining UTR variation may therefore provide further insight into the regulation of *ANK3* transcripts across neural cell types.

### Technical challenges

We selected *ANK3* as a case study partly because of its challenging characteristics: numerous alternatively spliced exons, many of which had low expression, expressed in different cell types. Our technical approach involving a combination of long read sequencing followed by FACS and ddPCR may appear complex compared to single-nuclei RNA sequencing (snRNAseq), but there are several good reasons for the choice: 1. snRNAseq would not have given an overview of the full-length transcripts as scRNAseq employs short reads; 2. it would not have been possible to measure the expression of alternative splice forms because scRNAseq methods only sequence the 5′ or 3′ end of genes; 3. in snRNAseq, a limited amount of sequencing is applied to each cell such that only the most highly expressed transcripts are detected (and only the most highly expressed of these are reliably measured); 4. without sorting, the cell type distribution would be very skewed towards the most abundant cells.

### Nanopore sequencing

We selected Oxford Nanopore sequencing because *ANK3* isoforms can be as long as 13 kbp (when incorporating the large neuronal exon) and there are long stretches of constitutive exons (Fig. 2e) which need to be read through by a read to determine how alternatively spliced exons are combined into transcripts. *ANK3* is a relatively lowly expressed gene and we aimed to characterize even its minor isoforms, so full-length amplification was required to obtain a sample where *ANK3* transcripts were dominant. To increase the likelihood of capturing transcripts expressed across the major neural cell types, we selected two regions with contrasting cellular composition: the corpus callosum enriched for oligodendrocytes and the frontal lobe enriched for neuronal cell bodies. As a result, our approach prevents the discovery of transcripts with novel TSSes but, based on previous work [19] and the expression of the constitutive splice patterns, no other TSSes appear to be active in the brain.

### Nuclei isolation and FACS

To investigate the expression of the alternatively spliced exons, we chose to isolate and sort nuclei instead of cells. To sort nuclei from neurons and oligodendrocytes, we chose the well-established markers neuronal nuclear protein (NeuN) and the oligodendrocyte transcription factor Olig2, respectively [55]. Recognizing the difficulty to sort nuclei from astrocytes, we used two published protocols as our starting point [56, 57], with the difference that our samples were frozen, instead of fresh brains, and we skipped the chemical glyoxal fixation step. Besides, during antibodies incubation we were careful to add 0.1% triton X-100 to the medium for easier access to SOX9 epitope, and 0.2 U/mL of RI during antibodies incubation and nuclei sorting to avoid RNA degradation. This enabled us to sort the three main brain cell types, although the yield of astrocytes was very low. The reasons for this are unclear, but possible explanations include: (i) low antibody affinity, (ii) epitope masking or degradation due to the nuclei isolation process, (iii) low abundance of the SOX9+ protein in the astrocytic nuclei. Another limitation of our FACS procedure is its limited cell type resolution. By using antibodies targeting nuclei specific sub-types of neurons [55], it would be possible to determine whether some of the neuronal exons have sub-type specific expression.

### Expression measures with ddPCR

We selected digital PCR as our method of expression measurement because of its high sensitivity and precision relative to qPCR. We carefully selected the endogenous control gene by ensuring that it had an expression level similar to *Ank3* and that it was stably expressed across individuals. However, in the process of measuring the gene’s absolute concentration in our cell sortings, we found that there is a systematic difference in expression across cell types (Fig. S11). This does not represent a problem for comparisons within cell types, but this is an interesting observation that is relevant to any measurement of expression across cell types. It is possible that other candidate endogenous controls display more stability than *Top1* across cell types, but any difference will need to be adjusted for in cross cell type comparisons. Some of this uncertainty could have been alleviated by using a panel of several reference genes, but scarcity of RNA material prevented us from running more ddPCR assays on all samples.

It is important to note that we did not pool our different cell sortings: each measurement is subject to both biological variation and technical variation throughout the protocol (not just technical variation of the ddPCR measurement). As a result, the statistical significance of our results takes into account these different sources of variation.

The large neuronal exon has a special status: it is one of the longest alternatively spliced exons in the human genome and its incorporation into the *ANK3* transcript doubles the size of the encoded protein. Its name suggests that the exon is specific to neurons, but it has already been shown that this exon is incorporated into transcripts in the heart [58] and our data suggests that it is also expressed in other neural cell types. This apparent non-specificity could be caused by poor quality sorting but this seems unlikely, since we obtain clear indications of cell type specificity for other exons (Fig. 5). We also find that most transcripts do not incorporate this exon irrespective of cell type in mouse (Fig. 4) which contrasts with humans where most neuronal transcripts incorporate some form of the neuronal exon [17].

### Conclusion

It is highly likely that polygenic diseases are caused by complex imbalances of specific transcripts of the associated genes in specific cell types [59]. The elucidation of a polygenic disease’s etiology will then require a comprehensive overview of all the transcripts of the associated genes and of the cell types in which they are expressed. In this work, we picked the *ANK3* gene as a challenging case study because it has numerous lowly-expressed alternatively spliced exons and low frequency variants with functional effects on specific isoforms have been associated with the disorder [17–19]. We showed how a combination of advanced methods enables us to obtain an overview of the gene’s isoforms and the cell type specificity of the alternatively spliced exons. These results provide valuable information about *ANK3* which has a central role in the biology of specific neural cell structures (nodes of Ranvier and the adjacent axoglial junctions) and has a well-established association with BD and schizophrenia. Our results also illustrate some of the general technical challenges involved in obtaining this information. In the future, more high-throughput methods need to be developed that address these technical challenges to enable the rapid characterization of the transcriptome of the hundreds of genes that are typically associated with prevalent polygenic pathologies.

## Supplementary information

Supplementary tables

Supplementary figures

## Data Availability

Long read data of full-length cDNA has been deposited in the NCBI’s SRA repository. Submission codes: SUB16123457 (human) and SUB16125599 (mouse).

## References

[1] Mountjoy E, Schmidt EM, Carmona M, Schwartzentruber J, Peat G, Miranda A, et al. An open approach to systematically prioritize causal variants and genes at all published human GWAS trait-associated loci. Nat Genet. 2021;53:1527–33. 10.1038/s41588-021-00945-5 PMC7611956 34711957

[2] Xiao X, Chang H, Li M. Molecular mechanisms underlying noncoding risk variations in psychiatric genetic studies. Mol Psychiatry. 2017;22:497–511. 10.1038/mp.2016.241 PMC5378805 28044063

[3] Li M, Li T, Xiao X, Chen J, Hu Z, Fang Y. Phenotypes, mechanisms and therapeutics: insights from bipolar disorder GWAS findings. Mol Psychiatry. 2022;27:2927–39. 10.1038/s41380-022-01523-9 35351989

[4] Kordeli E, Lambert S, Bennett V, Ankyrin G. A new ankyrin gene with neural-specific isoforms localized at the axonal initial segment and node of Ranvier. J Biol Chem. 1995;270:2352–9. 10.1074/jbc.270.5.2352 7836469

[5] Bennett V, Lorenzo DN. Spectrin- and ankyrin-based membrane domains and the evolution of vertebrates. Curr Top Membr. 2013;72:1–37. 10.1016/B978-0-12-417027-8.00001-5 24210426

[6] Nelson AD, Jenkins PM. The splice is right: ANK3 and the control of cortical circuits. Biol Psychiatry. 2016;80:263–5. 10.1016/j.biopsych.2016.06.006 PMC5103303 27468628

[7] Leterrier C, Potier J, Caillol G, Debarnot C, Rueda Boroni F, Dargent B. Nanoscale architecture of the axon initial segment reveals an organized and robust scaffold. Cell Rep. 2015;13:2781–93. 10.1016/j.celrep.2015.11.051 26711344

[8] Smith KR, Kopeikina KJ, Fawcett-Patel JM, Leaderbrand K, Gao R, Schürmann B, et al. Psychiatric risk factor ANK3/Ankyrin-G nanodomains regulate the structure and function of glutamatergic synapses. Neuron. 2014;84:399–415. 10.1016/j.neuron.2014.10.010 PMC4223651 25374361

[9] Yoon S, Myczek K, Penzes P. cAMp signaling–mediated phosphorylation of diacylglycerol lipase α regulates interaction with Ankyrin-G and dendritic spine morphology. Biol Psychiatry. 2021;90:263–74. 10.1016/j.biopsych.2021.03.023 PMC8384113 34099188

[10] Chang K-J, Zollinger DR, Susuki K, Sherman DL, Makara MA, Brophy PJ, et al. Glial ankyrins facilitate paranodal axoglial junction assembly. Nat Neurosci. 2014;17:1673–81. 10.1038/nn.3858 PMC4260775 25362471

[11] Ding X, Wu Y, Rodriguez V, Ricco E, Okoh JT, Liu Y, et al. Age-dependent regulation of axoglial interactions and behavior by oligodendrocyte AnkyrinG. Nat Commun. 2024;15:10865 10.1038/s41467-024-55209-7. PMC11686269 39738113

[12] Pgc. Large-scale genome-wide association analysis of bipolar disorder identifies a new susceptibility locus near ODZ4. Nat Genet. 2011;43:977–83. 10.1038/ng.943 PMC3637176 21926972

[13] Mühleisen TW, Leber M, Schulze TG, Strohmaier J, Degenhardt F, Treutlein J, et al. Genome-wide association study reveals two new risk loci for bipolar disorder. Nat Commun. 2014;5:3339. 10.1038/ncomms4339 24618891

[14] Stahl EA, Breen G, Forstner AJ, McQuillin A, Ripke S, Trubetskoy V, et al. Genome-wide association study identifies 30 loci associated with bipolar disorder. Nat Genet. 2019;51:793–803. 10.1038/s41588-019-0397-8 PMC6956732 31043756

[15] Mullins N, Forstner AJ, O’Connell KS, Coombes B, Coleman JRI, Qiao Z, et al. Genome-wide association study of more than 40,000 bipolar disorder cases provides new insights into the underlying biology. Nat Genet. 2021;53:817–29. 10.1038/s41588-021-00857-4 PMC8192451 34002096

[16] O’Connell KS, Koromina M, van der Veen T, Boltz T, David FS, Yang JMK, et al. Genomics yields biological and phenotypic insights into bipolar disorder. Nature 2025;639:968–75. 10.1038/s41586-024-08468-9 PMC12163093 39843750

[17] Rueckert EH, Barker D, Ruderfer D, Bergen SE, O’Dushlaine C, Luce CJ, et al. Cis-acting regulation of brain-specific ANK3 gene expression by a genetic variant associated with bipolar disorder. Mol Psychiatry. 2013;18:922–9. 10.1038/mp.2012.104 PMC3856665 22850628

[18] Hughes T, Hansson L, Sønderby IE, Athanasiu L, Zuber V, Tesli M, et al. A loss-of-function variant in a minor isoform of ANK3 protects against bipolar disorder and schizophrenia. Biol Psychiatry. 2016;80:323–30. 10.1016/j.biopsych.2015.09.021 26682468

[19] Hughes T, Sønderby IE, Polushina T, Hansson L, Holmgren A, Athanasiu L, et al. Elevated expression of a minor isoform of ANK3 is a risk factor for bipolar disorder. Transl Psychiatry. 2018;8:210. 10.1038/s41398-018-0175-x PMC6175894 30297702

[20] Athanasiu L, Mattingsdal M, Kähler AK, Brown A, Gustafsson O, Agartz I, et al. Gene variants associated with schizophrenia in a Norwegian genome-wide study are replicated in a large European cohort. J Psychiatr Res. 2010;44:748–53. 10.1016/j.jpsychires.2010.02.002 PMC3224994 20185149

[21] Pgc PGC. Genome-wide association study identifies five new schizophrenia loci. Nat Genet. 2011;43:969–76. 10.1038/ng.940 PMC3303194 21926974

[22] Yuan A, Yi Z, Wang Q, Sun J, Li Z, Du Y, et al. ANK3 as a risk gene for schizophrenia: new data in Han Chinese and meta analysis. Am J Med Genet B Neuropsychiatr Genet. 2012;159B:997–1005. 10.1002/ajmg.b.32112 23109352

[23] Yang R, Walder-Christensen KK, Lalani S, Yan H, García-Prieto ID, Álvarez S, et al. Neurodevelopmental mutation of giant ankyrin-G disrupts a core mechanism for axon initial segment assembly. Proc Natl Acad Sci USA. 2019;116:19717–26. 10.1073/pnas.1909989116 PMC6765234 31451636

[24] Panagiotakaki E, Tiziano FD, Mikati MA, Vijfhuizen LS, Nicole S, Lesca G, et al. Exome sequencing of ATP1A3-negative cases of alternating hemiplegia of childhood reveals SCN2A as a novel causative gene. Eur J Hum Genet. 2023;32:224–31. 10.1038/s41431-023-01489-4 PMC10853263 38097767

[25] Kloth K, Denecke J, Hempel M, Johannsen J, Strom TM, Kubisch C, et al. First de novo ANK3 nonsense mutation in a boy with intellectual disability, speech impairment and autistic features. Eur J Med Genet. 2017;60:494–8. 10.1016/j.ejmg.2017.07.001 28687526

[26] Kloth K, Lozic B, Tagoe J, Hoffer MJV, Van der Ven A, Thiele H, et al. ANK3 related neurodevelopmental disorders: expanding the spectrum of heterozygous loss-of-function variants. Neurogenetics. 2021;22:263–9. 10.1007/s10048-021-00655-4 PMC8426245 34218362

[27] Bi C, Wu J, Jiang T, Liu Q, Cai W, Yu P, et al. Mutations of ANK3 identified by exome sequencing are associated with autism susceptibility. Hum Mutat. 2012;33:1635–8. 10.1002/humu.22174 22865819

[28] Iqbal Z, Vandeweyer G, van der Voet M, Waryah AM, Zahoor MY, Besseling JA, et al. Homozygous and heterozygous disruptions of ANK3: at the crossroads of neurodevelopmental and psychiatric disorders. Hum Mol Genet. 2013;22:1960–70. 10.1093/hmg/ddt043 23390136

[29] Furia F, Levy AM, Theunis M, Bamshad MJ, Bartos MN, Bijlsma EK et al. The phenotypic and genotypic spectrum of individuals with mono- or biallelic ANK3 variants. *Clin Genet* 2024; **18**. 10.1111/CGE.14587. PMC11444875 38988293

[30] van der Werf IM, Jansen S, de Vries PF, Gerstmans A, van de Vorst M, Van Dijck A, et al. Overrepresentation of genetic variation in the AnkyrinG interactome is related to a range of neurodevelopmental disorders. Eur J Hum Genet. 2020;28:1726–33. 10.1038/s41431-020-0682-0 PMC7785003 32651551

[31] Ho TS-Y, Zollinger DR, Chang K-J, Xu M, Cooper EC, Stankewich MC, et al. A hierarchy of ankyrin-spectrin complexes clusters sodium channels at nodes of ranvier. Nat Neurosci. 2014;17:1664–72. 10.1038/nn.3859 PMC4271271 25362473

[32] Jenkins PM, Kim N, Jones SL, Tseng WC, Svitkina TM, Yin HH, et al. Giant ankyrin-G: a critical innovation in vertebrate evolution of fast and integrated neuronal signaling. Proceedings of the National Academy of Sciences. 2015;112:957–64. 10.1073/pnas.1416544112 PMC4313853 25552556

[33] Won E, Kim Y-K. An oldie but goodie: lithium in the treatment of bipolar disorder through neuroprotective and neurotrophic mechanisms. Int J Mol Sci. 2017;18:2679 10.3390/ijms18122679. PMC5751281 29232923

[34] Rybakowski JK. Lithium in neuropsychiatry: a 2010 update. World J Biol Psychiatry. 2011;12:340–8. 10.3109/15622975.2011.559274 21361856

[35] Piguel NH, Sanders SS, De Simone FI, Martin-de-Saavedra MD, McCoig E, Dionisio LE, et al. Palmitoylation controls the stability of 190 kDa ankyrin-G in dendritic spines and is regulated by ZDHHC8 and lithium. Front Mol Neurosci. 2023;16:1144066. 10.3389/fnmol.2023.1144066 PMC10031057 36969554

[36] Leussis MP, Madison JM, Petryshen TL. Ankyrin 3: genetic association with bipolar disorder and relevance to disease pathophysiology. Biol Mood Anxiety Disord. 2012;2:18. 10.1186/2045-5380-2-18 PMC3492013 23025490

[37] Piguel NH, Yoon S, Gao R, Horan KE, Garza JC, Petryshen TL, et al. Lithium rescues dendritic abnormalities in Ank3 deficiency models through the synergic effects of GSK3β and cyclic AMP signaling pathways. Neuropsychopharmacology. 2022;48:1000–10. 10.1038/s41386-022-01502-2 PMC10209204 36376465

[38] Caballero-Florán RN, Dean KP, Nelson AD, Min L, Jenkins PM. Lithium restores inhibitory function and neuronal excitability through GSK-3β inhibition in a bipolar disorder-associated Ank3 variant mouse model. Neuropharmacology 2025;279:110649. 10.1016/j.neuropharm.2025.110649 PMC12455633 40849089

[39] Wang G, Aguado M, Spear MA, Alphs L, Chen C, Huang H, et al. ANK3 as a novel genetic biomarker for liafensine in treatment-resistant depression: The ENLIGHTEN randomized clinical trial. JAMA Psychiatry. 2025. 10.1001/jamapsychiatry.2025.2416. PMC12423948 40928787

[40] Shen W, Le S, Li Y, Hu F. SeqKit: a cross-platform and ultrafast toolkit for FASTA/Q file manipulation. PLoS One. 2016;11:e0163962. 10.1371/journal.pone.0163962 PMC5051824 27706213

[41] Li H. Minimap2: pairwise alignment for nucleotide sequences. Bioinformatics. 2018;34:3094–3100. 10.1093/bioinformatics/bty191 PMC6137996 29750242

[42] Li H, Handsaker B, Wysoker A, Fennell T, Ruan J, Homer N, et al. The sequence Alignment/Map format and SAMtools. Bioinformatics. 2009;25:2078–9. 10.1093/bioinformatics/btp352 PMC2723002 19505943

[43] Robinson JT, Thorvaldsdóttir H, Winckler W, Guttman M, Lander ES, Getz G, et al. Integrative genomics viewer. Nat Biotechnol. 2011;29:24–26. 10.1038/nbt.1754 PMC3346182 21221095

[44] Dyer SC, Austine-Orimoloye O, Azov AG, Barba M, Barnes I, Barrera-Enriquez VP, et al. Ensembl 2025. Nucleic Acids Res. 2025;53:D948–D957. 10.1093/nar/gkae1071 PMC11701638 39656687

[45] Goldfarb T, Kodali VK, Pujar S, Brover V, Robbertse B, Farrell CM, et al. NCBI RefSeq: reference sequence standards through 25 years of curation and annotation. Nucleic Acids Res. 2025;53:D243–D257. 10.1093/nar/gkae1038 PMC11701664 39526381

[46] Tang AD, Soulette CM, van Baren MJ, Hart K, Hrabeta-Robinson E, Wu CJ, et al. Full-length transcript characterization of SF3B1 mutation in chronic lymphocytic leukemia reveals downregulation of retained introns. Nat Commun. 2020;11:1438. 10.1038/s41467-020-15171-6 PMC7080807 32188845

[47] Diessler S, Jan M, Emmenegger Y, Guex N, Middleton B, Skene DJ, et al. A systems genetics resource and analysis of sleep regulation in the mouse. PLOS Biology. 2018;16:e2005750. 10.1371/journal.pbio.2005750 PMC6085075 30091978

[48] Robinson MD, McCarthy DJ, Smyth GK. edgeR: a bioconductor package for differential expression analysis of digital gene expression data. Bioinformatics. 2010;26:139–40. 10.1093/bioinformatics/btp616 PMC2796818 19910308

[49] Rydbirk R, Folke J, Winge K, Aznar S, Pakkenberg B, Brudek T. Assessment of brain reference genes for RT-qPCR studies in neurodegenerative diseases. Sci Rep. 2016;6:37116. 10.1038/srep37116 PMC5112547 27853238

[50] Holmgren A, Hansson L, Bjerkaas-Kjeldal K, Impellizzeri AAR, Gilfillan GD, Djurovic S, et al. Mapping the expression of an ANK3 isoform associated with bipolar disorder in the human brain. Translational Psychiatry. 2022;12:1–8. 10.1038/s41398-022-01784-6 PMC8799726 35091539

[51] Sundaram VK, Sampathkumar NK, Massaad C, Grenier J. Optimal use of statistical methods to validate reference gene stability in longitudinal studies. PLoS One. 2019;14:e0219440. 10.1371/journal.pone.0219440 PMC6650036 31335863

[52] Ritchie ME, Phipson B, Wu D, Hu Y, Law CW, Shi W, et al. limma powers differential expression analyses for RNA-sequencing and microarray studies. Nucleic Acids Res. 2015;43:e47. 10.1093/nar/gkv007 PMC4402510 25605792

[53] Taylor SC, Laperriere G, Germain H. Droplet digital PCR versus qPCR for gene expression analysis with low abundant targets: from variable nonsense to publication quality data. Sci Rep. 2017;7:2409. 10.1038/s41598-017-02217-x PMC5445070 28546538

[54] Kumar M, Michael S, Alvarado-Valverde J, Zeke A, Lazar T, Glavina J, et al. ELM-the Eukaryotic Linear Motif resource-2024 update. Nucleic Acids Res. 2024;52:D442–D455. 10.1093/nar/gkad1058 PMC10767929 37962385

[55] Wiseman JA, Dragunow M, Park TI-H. Cell type-specific nuclei markers: the need for human brain research to go nuclear. Neuroscientist. 2023;29:41–61. 10.1177/10738584211037351 34459315

[56] Sun W, Cornwell A, Li J, Peng S, Osorio MJ, Aalling N, et al. SOX9 is an astrocyte-specific nuclear marker in the adult brain outside the neurogenic regions. J Neurosci. 2017;37:4493–507. 10.1523/JNEUROSCI.3199-16.2017 PMC5413187 28336567

[57] Labarta-Bajo L, Deng J, Contreras M, Allen NJ. Protocol for the purification and transcriptomic analysis of mouse astrocytes using GFAT. STAR Protoc. 2023;4:102599. 10.1016/j.xpro.2023.102599 PMC10522990 37742178

[58] Cavus O, Williams J, Musa H, El Refaey M, Gratz D, Shaheen R, et al. Giant ankyrin-G regulates cardiac function. J Biol Chem. 2021;296:100507. 10.1016/j.jbc.2021.100507 PMC8040283 33675749

[59] Holmgren A, Akkouh I, O’Connell KS, Osete JR, Bjørnstad P, Djurovic S, et al. Bipolar patients display stoichiometric imbalance of gene expression in post-mortem brain samples. Mol Psychiatry. 2024;29:1128–38. 10.1038/s41380-023-02398-0 PMC11176081 38351171

[60] Chang K-J, Rasband MN. Chapter five – excitable domains of myelinated nerves: axon initial segments and nodes of ranvier. In: *Current Topics in Membranes*. Waltham, MA: Academic Press. 2013; pp. 159–92. 10.1016/B978-0-12-417027-8.00005-2 24210430

[61] Huang CY-M, Zhang C, Ho TS-Y, Oses-Prieto J, Burlingame AL, Lalonde J, et al. αII spectrin forms a periodic cytoskeleton at the axon initial segment and is required for nervous system function. J Neurosci. 2017;37:11311–22. 10.1523/JNEUROSCI.2112-17.2017 PMC5700417 29038240

[62] Blanchette M, Kent WJ, Riemer C, Elnitski L, Smit AFA, Roskin KM, et al. Aligning multiple genomic sequences with the threaded blockset aligner. Genome Res. 2004;14:708–15. 10.1101/gr.1933104 PMC383317 15060014

