# Supplementary material for: Surveying alternative transcripts of disease-associated genes and their cell type specificity – a case study with ANK3
Source: Transl Psychiatry. 2026 Oct 1;16:531. doi: 10.1038/s41398-026-04412-9 (PMC13646255; doi:10.1038/s41398-026-04412-9)
Supplement: Supplementary file 1 [file 41398_2026_4412_MOESM2_ESM.pdf]

Supplementary materials

Oxford Nanopore full-length cDNA sequencing

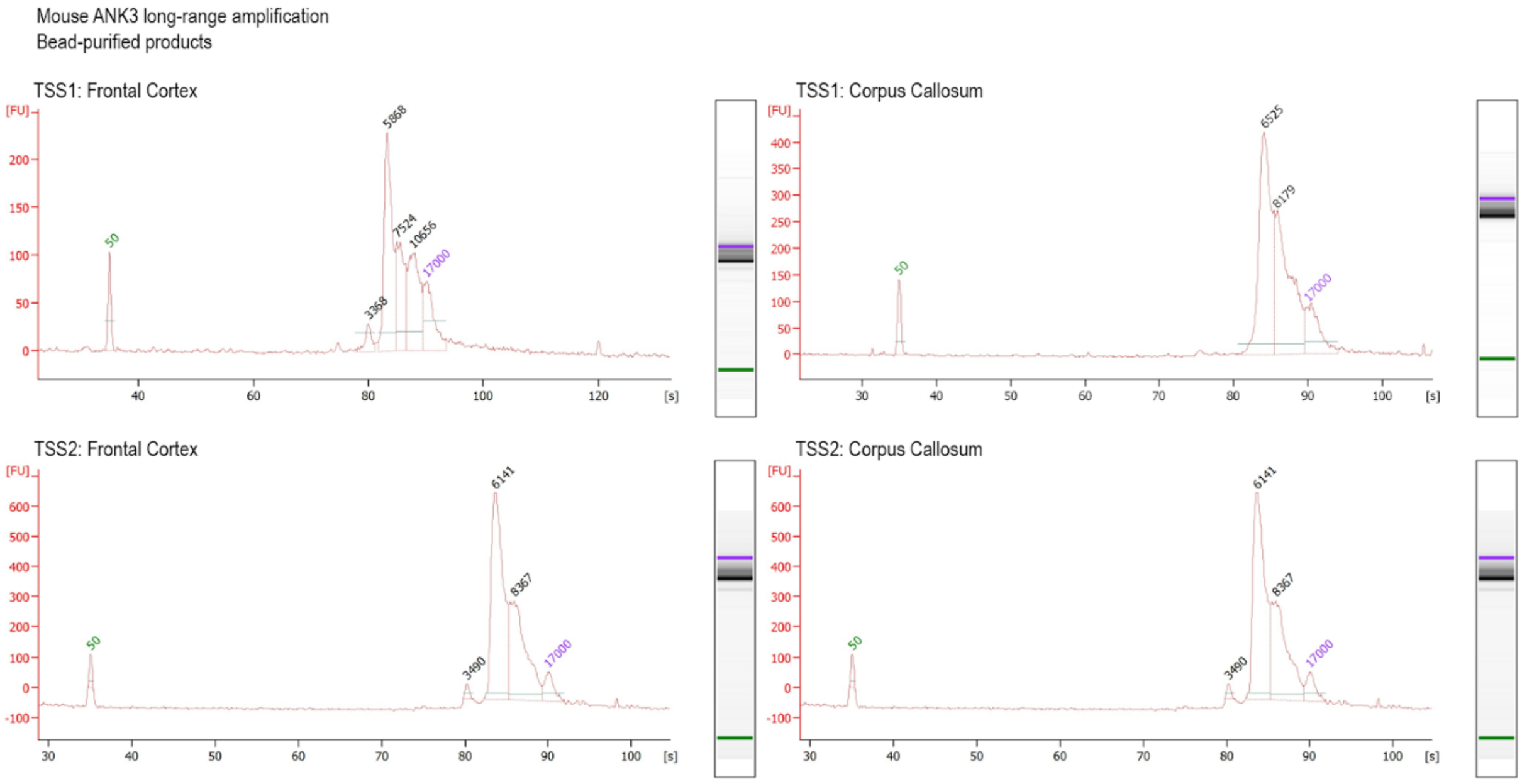

Figure S1: Mouse *Ank3* long-range amplification products measured on a BioAnalyzer. These readouts confirm that we successfully amplified *Ank3* isoforms of different lengths.

Nanopore sequencing reads in IGV

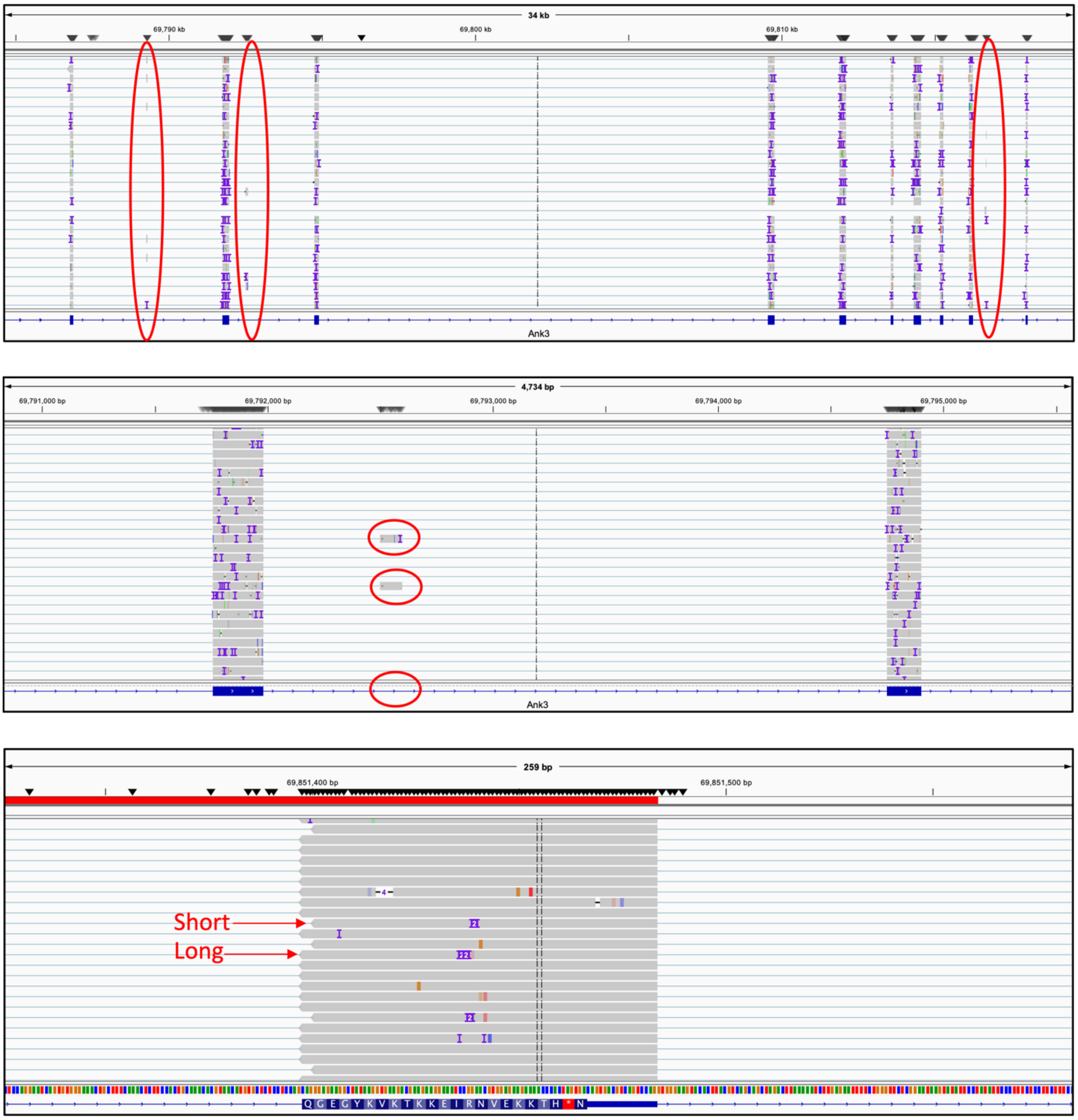

Figure S2: Oxford Nanopore reads mapped to mouse genomic sequence of *Ank3* (displayed in the IGV browser)

In each panel, the upper part of each image contains the mapped Nanopore reads: one read per line, with the thick part of read representing read sequence (mapping to the exons) and the thin part representing a gap in the alignment (corresponding to the introns). The colored bases within the reads are insertion/deletion/mismatch errors in the read which are common with Oxford Nanopore sequencing. The lower part of each image displays part of the RefSeq exon structure of *Ank3*.

Top panel: overview of a region containing many exons with 3 locations (circled in red) where an alternatively spliced exon is observed in the Nanopore reads, but where the RefSeq annotation does not contain the exon.

Middle panel: a close up of one of the alternatively spliced exons from the top panel. Exonic sequence of two reads containing the exon and the missing exon annotation in RefSeq (circled in red).

Bottom panel: a constitutive exon with the Nanopore reads displaying two alternative lengths (short and long), but where the RefSeq annotation only contains the longer of the two forms.

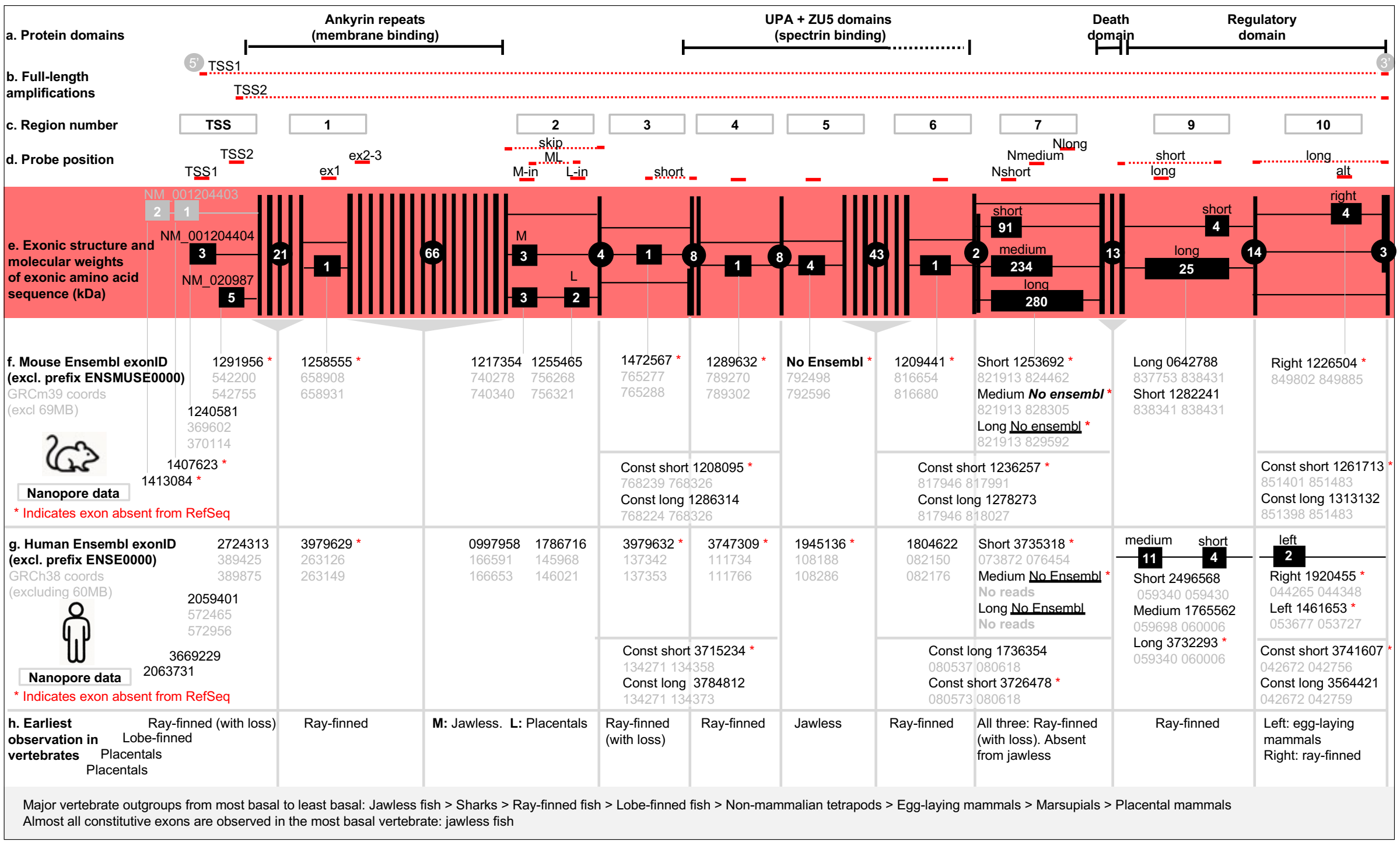

Figure S3: Full-length transcript Nanopore sequencing and ddPCR probes

a. Protein domains: as annotated in Ensembl with additions from [5].

b. Full-length amplifications: primer positions.

c. Region number: numbering of alternatively spliced regions. Region 8 is deliberately absent (it was putative, but not confirmed). In each region, the skipping the alternatively spliced exon is usually the most highly expressed splice pattern.

d. Expression probe position: schematic of ddPCR probe positions.

e. Exonic structure and molecular weights: black vertical bars depict constitutive exons. Numbers displayed in alternatively spliced exons or in circles over constitutive exons are the molecular weights of the encoded peptide and are broadly proportional to the amino acid length.

f. Mouse and **g.** human exon details: Details of the exons observed in the long-read sequencing data.

- In black text, the Ensembl exonID (stripped of prefix ENSMUSE0000 for mouse and stripped of prefix ENSE0000 for human), beneath each ID in grey are the coordinates of the exon if it was observed in the long-read sequencing data. When an exon was observed in the read data but is not registered in Ensembl, the ID is "No Ensembl". When an exon was not observed in the read data, the position is "No reads".
- A red * indicates and exon that is not registered in RefSeq.
- There are 3 constitutive exons with both a short and a longer form between regions 3 and 4, 6 and 7, and at the end of region 10 (which are 3, 15 and 36 nucleotides longer).
- The figure only includes protein coding exons (exons that consist of 100% untranslated region are not represented). However, exon coordinates for 5' and 3' exons are for the whole exon (including any UTR region).

h. Earliest observation in vertebrates: name of most basal vertebrate outgroup in which exon sequence is conserved, inferred from comparative genomics track from the UCSC Genome: 100 vertebrates genomes aligned with MultiZ.[62]. See supplementary materials for details.

Isoform build and quantification with FLAIR

ANK3 isoform analysis: We employed FLAIR (Tang et al. 2020) (1.6, running in Ubuntu 20.04 and Python 3.7.12) to define and analyze the isoforms of *ANK3* in our sample files. FLAIR was specifically developed for use with long-read technologies with high base-calling and indel rates. However, FLAIR may face some challenges in analyzing a very large gene with long stretches of constitutive exons (not alternatively spliced), such as the ankyrin repeats in *ANK3*. The FLAIR software includes several modules, three of which played a crucial role in our analysis: flair-correct, flair-collapse, and flair-quantify. The 'flair-correct' module corrects misaligned splice sites. The 'flair-collapse' module defines high-confidence isoforms and the 'flair-quantify' module generates an isoform-by-sample count file.

Synthetic gene injection: FLAIR detected most of the transcripts that we observed in the human data. However, some structurally distinct isoforms, that were observed in IGV alignments, were not detected by FLAIR. These were typically isoforms with low expression. To remedy this, we constructed eight synthetic *ANK3* gene models (based on the Refseq transcript NM_001204404.2) which included the splice patterns observed in the IGV alignments but missed by FLAIR and introduced them into the GTF file that is input to FLAIR. With this updated GTF file, FLAIR detected all but two of the transcripts observed in the data. FLAIR also failed to identify all the alternative splice forms of the large neuronal exon. This complex issue was solved by manually inspection and counting of the read alignments in IGV rather than making more adaptations to FLAIR. In the mouse data, two splice patterns were observed in the read alignments in IGV, but missed by FLAIR, and the issue was resolved in the same way as in humans by augmenting the GTF file with two synthetic gene models.

FLAIR output data: To visually summarize *ANK3* transcripts and their quantification, the FLAIR data output was imported and analyzed in Rstudio (R 4.12). We imported the isoform track data with the rtracklayer package (Lawrence, Gentleman, and Carey 2009) (1.54.0) and isoform count data from flair-quantify as a delimited text file. We rescaled and plotted the isoform structure with ggtranscript (Gustavsson et al. 2022) (3.3.5) and ggplot2 (Wickham n.d.) (3.3.5). We used GenomicAlignments (Lawrence et al. 2013) (1.30.0) to search for isoforms containing specific gene sequences (such as neuronal exon variations) as you could input a start and an end coordinate for a particular exon and get all the transcript names that contained this feature and then with cross referencing the count file FLAIR output, we were able to see how many isoforms with a particular feature there was together with how many counts that feature was attributed. Transcripts that matched to a published annotation were similarly annotated by FLAIR whereas all newly generated transcripts were given generated names by FLAIR's internal programming.

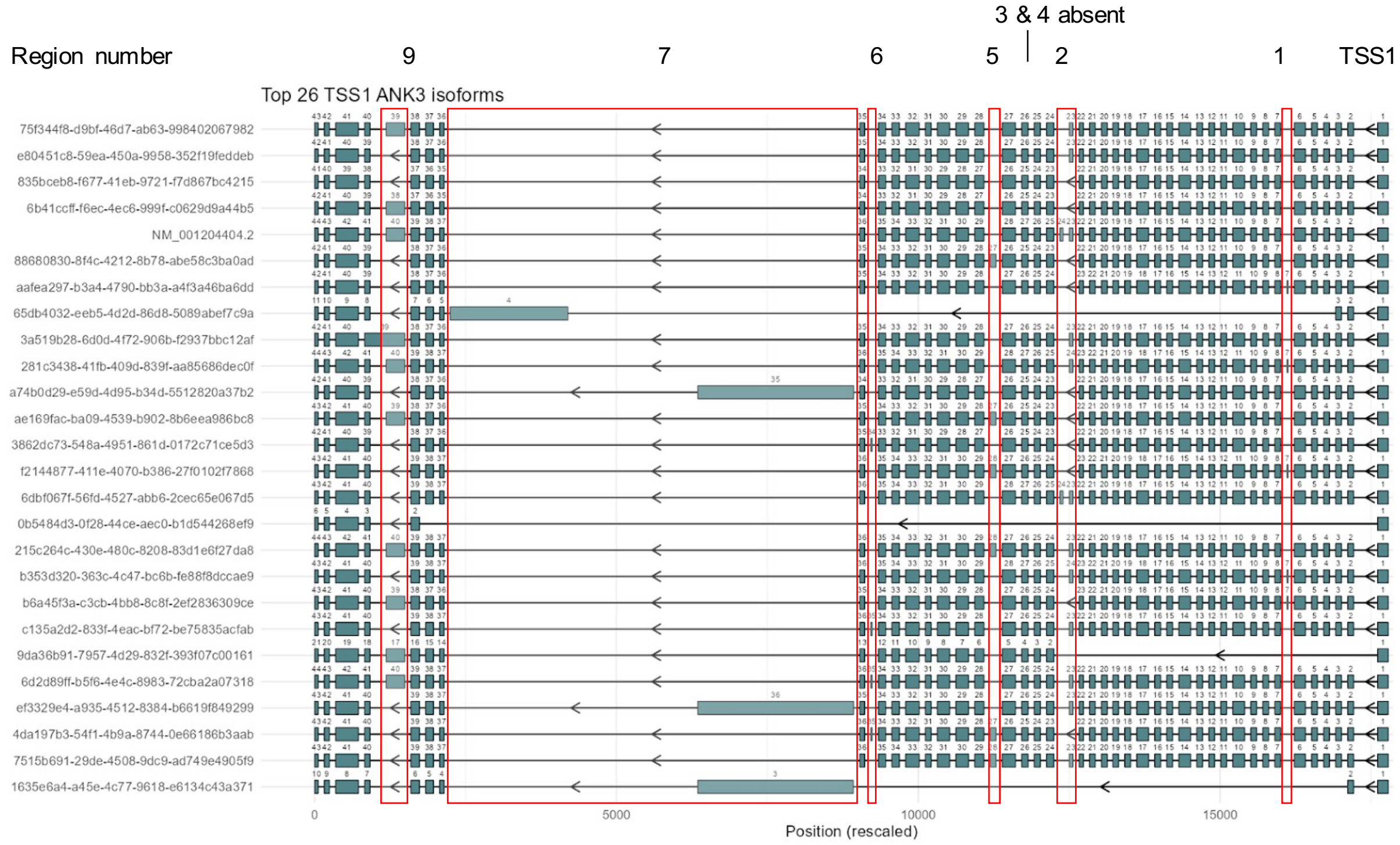

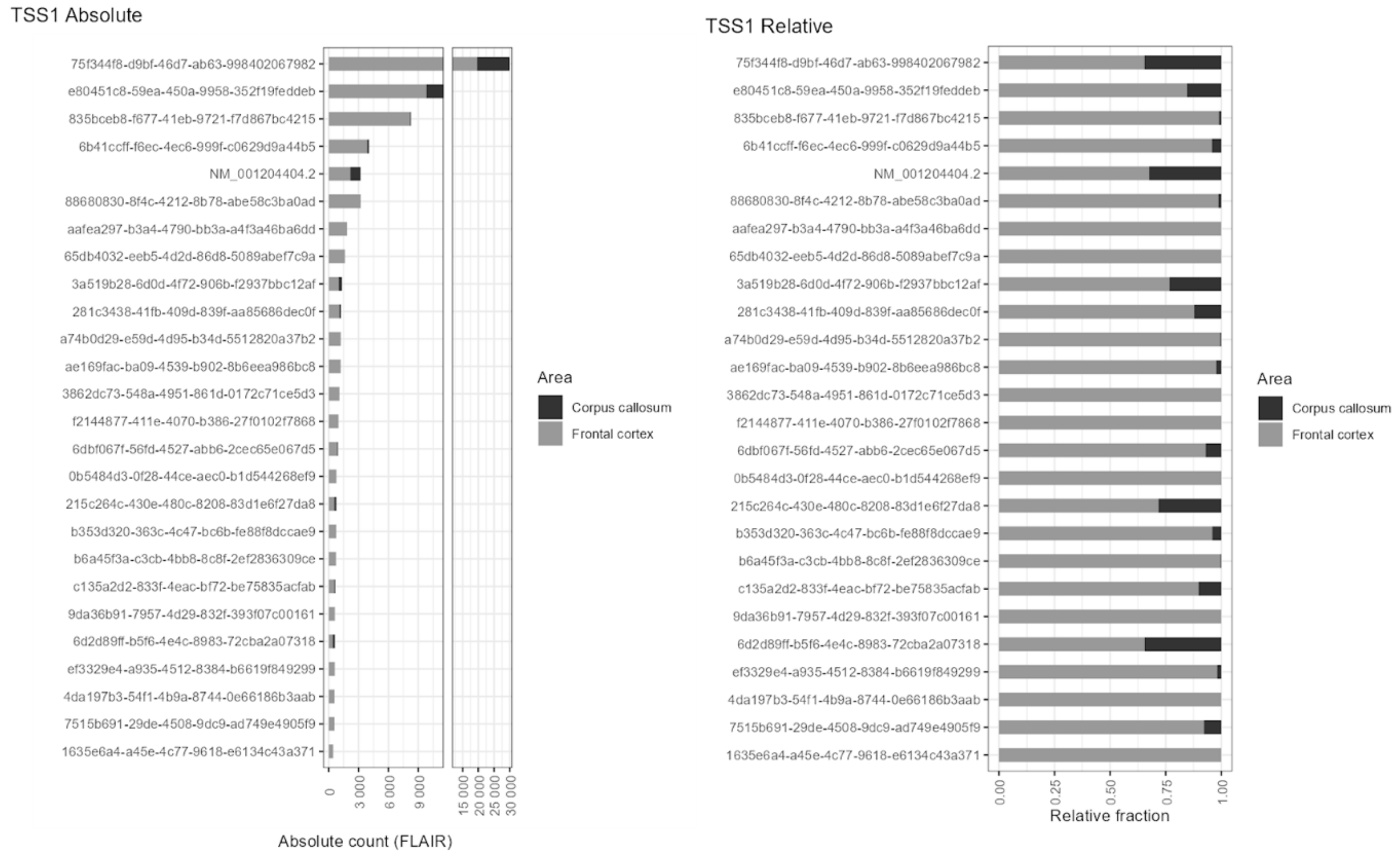

Figure S4: **Human** isoforms detected transcribed from **TSS1**

Top panel: 26 most abundant isoforms estimated by the FLAIR prediction pipeline

Lower panel: Abundances measured as raw absolute counts and relative fraction (in corpus callosum vs. frontal cortex)

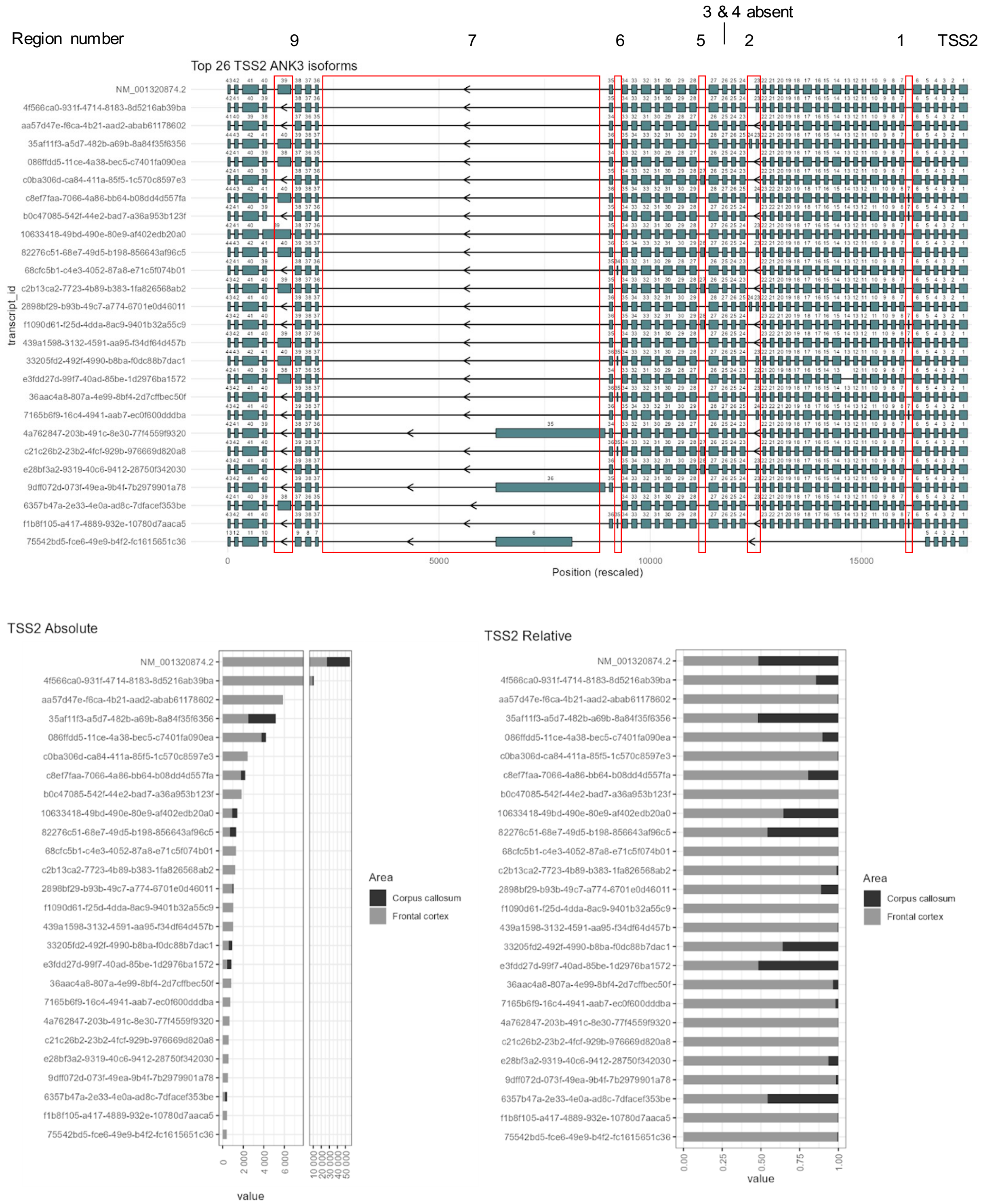

Figure S5: **Human** isoforms detected transcribed from **TSS2**
Top panel: 26 most abundant isoforms estimated by the FLAIR prediction pipeline
Lower panel: Abundances measured as raw absolute counts and relative fraction (in corpus callosum vs. frontal cortex)

Mouse full-length transcripts

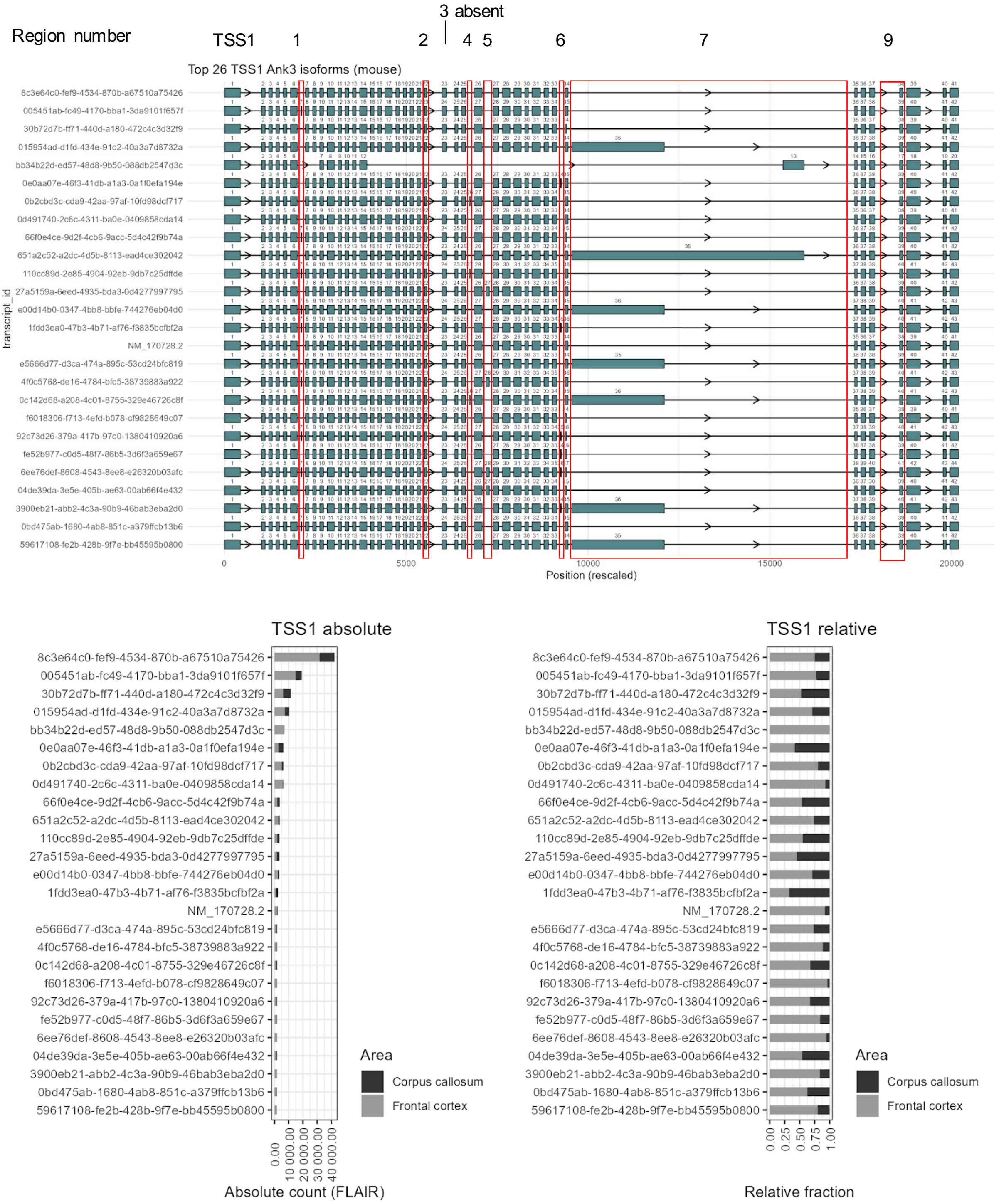

Figure S6: **Mouse** isoforms detected transcribed from **TSS1**
Top panel: 26 most abundant isoforms estimated by the FLAIR prediction pipeline
Lower panel: Abundances measured as raw absolute counts and relative fraction (in corpus callosum vs. frontal cortex)

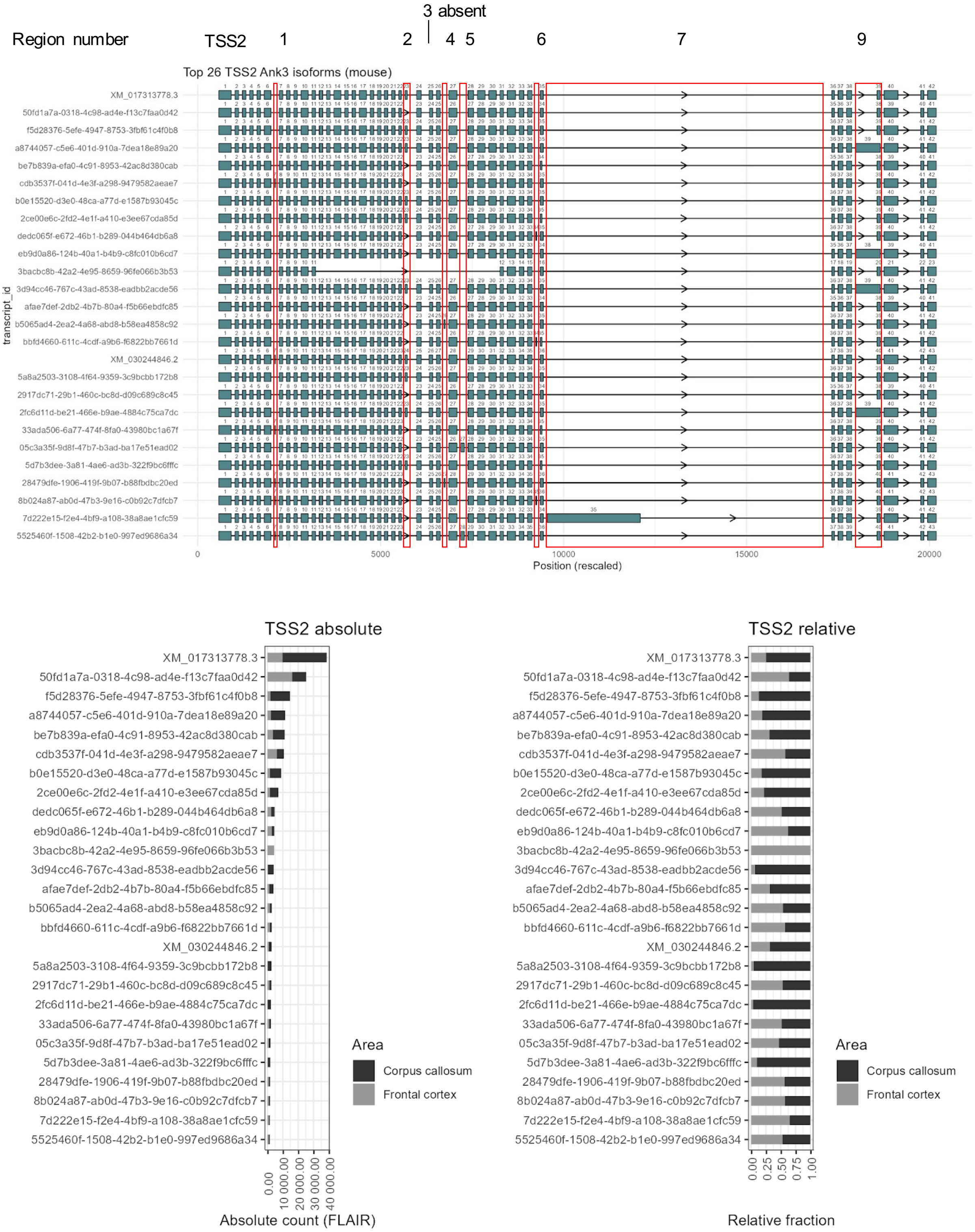

Figure S7: **Mouse** isoforms detected transcribed from **TSS2**

Top panel: 26 most abundant isoforms estimated by the FLAIR prediction pipeline

Lower panel: Abundances measured as raw absolute counts and relative fraction (in corpus callosum vs. frontal cortex)

Evolutionary annotation of alternatively spliced exons

We used a comparative genomics track from the UCSC Genome browser to infer the evolutionary origin of the alternatively spliced exons: the 100 vertebrates genomes aligned with MultiZ.(Blanchette et al. 2004). The major vertebrate species groups (from most to least basal) are as follows with the most basal species for the group in the Blanchette dataset in parenthesis. Note that no shark species is present in the dataset: Jawless fish (lamprey) > Sharks > Ray-finned fish (spotted gar) > Lobe-finned (coelecanth) > Non-mammalian tetrapods (X. tropicalis) > Egg-laying mammals (platypus) > Marsupials (opposum) > Placental mammals (armadillo). For each exon, we record the most basal outgroup (in the dataset) in which the exon sequence is observed. This means that the sequence is at least as old as that outgroup but, because only a selection of 100 species were sequenced and because sequence can also be lost from a genome, the sequence may have evolved earlier. For example, exons marked as "Ray-finned fish" may be present in sharks (but we lack sequence evidence for this as shark data is missing) or may have evolved as far back as jawless fish (but with sequence loss having occurred in the lamprey, so that it is not observed in the dataset).

Nuclei isolation and FACS

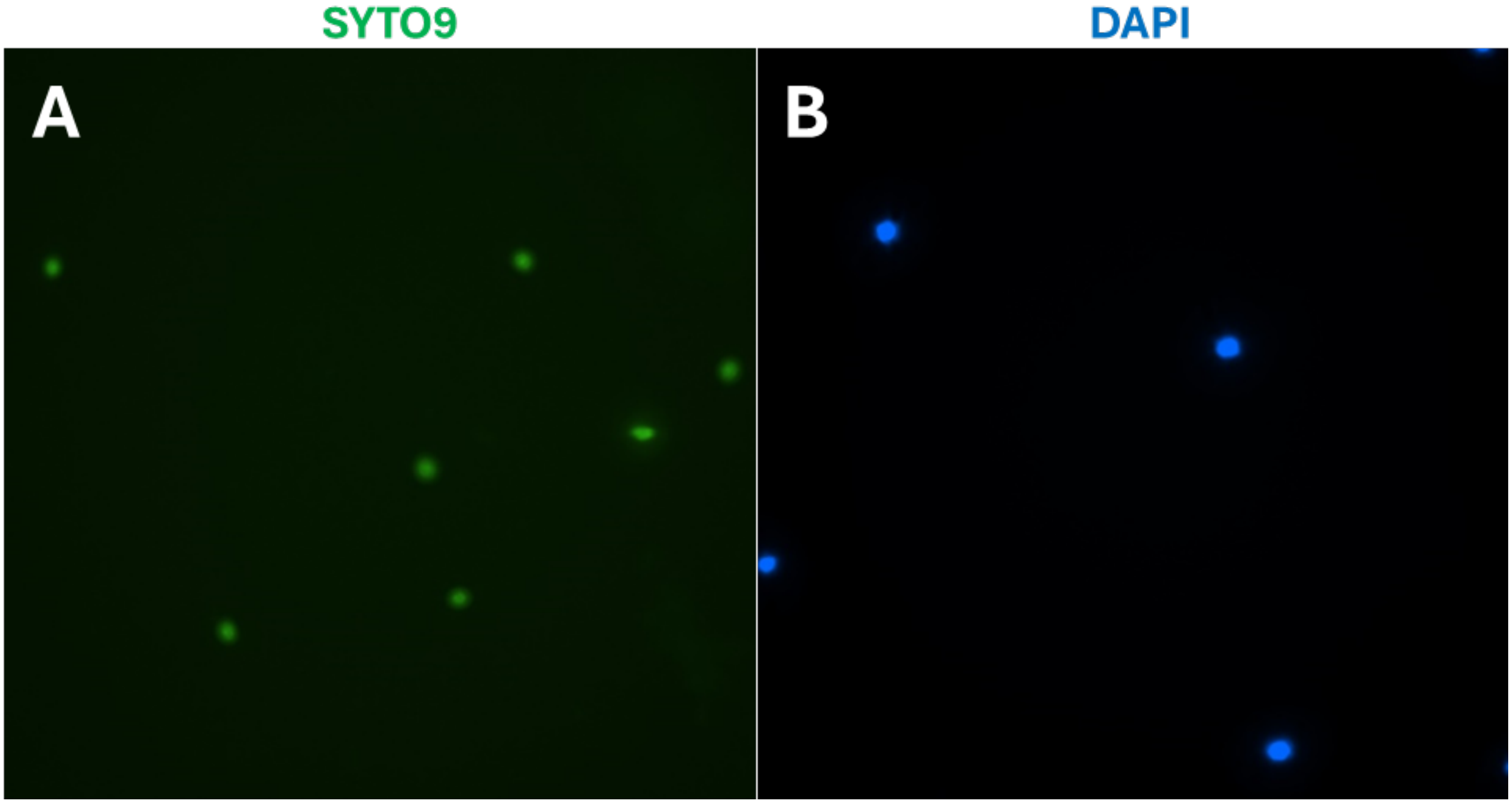

Figure S8. Isolated nuclei imaging. **A.** Nuclei stained with the RNA marker SYTO9 (green) and **B.** the DNA marker DAPI (blue). Images taken with 40X magnification. Images confirm presence of intact nuclei containing both DNA and RNA.

Sample acquisition and cell sorting

Sample acquisition and cell sorting was conducted on a BD FACSMelody™ cell sorter powered with the Chorus software (BD Biosciences) and equipped with a 100 µm nozzle and configured with three lasers; violet (405 nm), blue (488 nm), red (640 nm) with in total of 9 fluorescence detectors. Nuclei were identified in the 448/45 detector in a pulse area histogram of DAPI, and single nuclei were further selected in a dotplot of the pulse width of DAPI versus pulse area of forward scatter (FSC-A). Nuclei derived from neurons, oligodendrocytes, and astrocytes were identified in a counterplot of pulse area of anti-NeuN AF488 versus pulse area of anti-Olig2 AF647 and pulse area of anti-SOX9 PE, respectively. Fluorescence-minus-one (FMO) controls were used to identify nuclei subpopulations derived from oligodendrocytes, astrocytes and neurons prior to sorting. Purity checks on sorted populations were performed to check sorting efficiency. To avoid nuclei sticking to the tube when sorted, nuclei were sorted into Eppendorf® Protein LoBind tubes (Eppendorf , #003010811). Nuclei were sorted into dPBS buffer supplemented with 0.2 U/mL RI and then spun down immediately after sorting and the pellet was snap frozen on dry ice. Frozen pellets from the NeuN+, Olig2+, SOX9+, and SOX9- subpopulations of nuclei were lysed with 250 µL of RLT buffer (component of the RNeasy Micro kit, Qiagen, #74004) supplemented with 10% β-mercaptoethanol, except the SOX9+ fraction, which had lower number of nuclei and was lysed with 100 µL of the buffer. Samples were again snap frozen in dry ice and stored at -80°C until the day of RNA extraction. We performed a total of four FACS sortings.

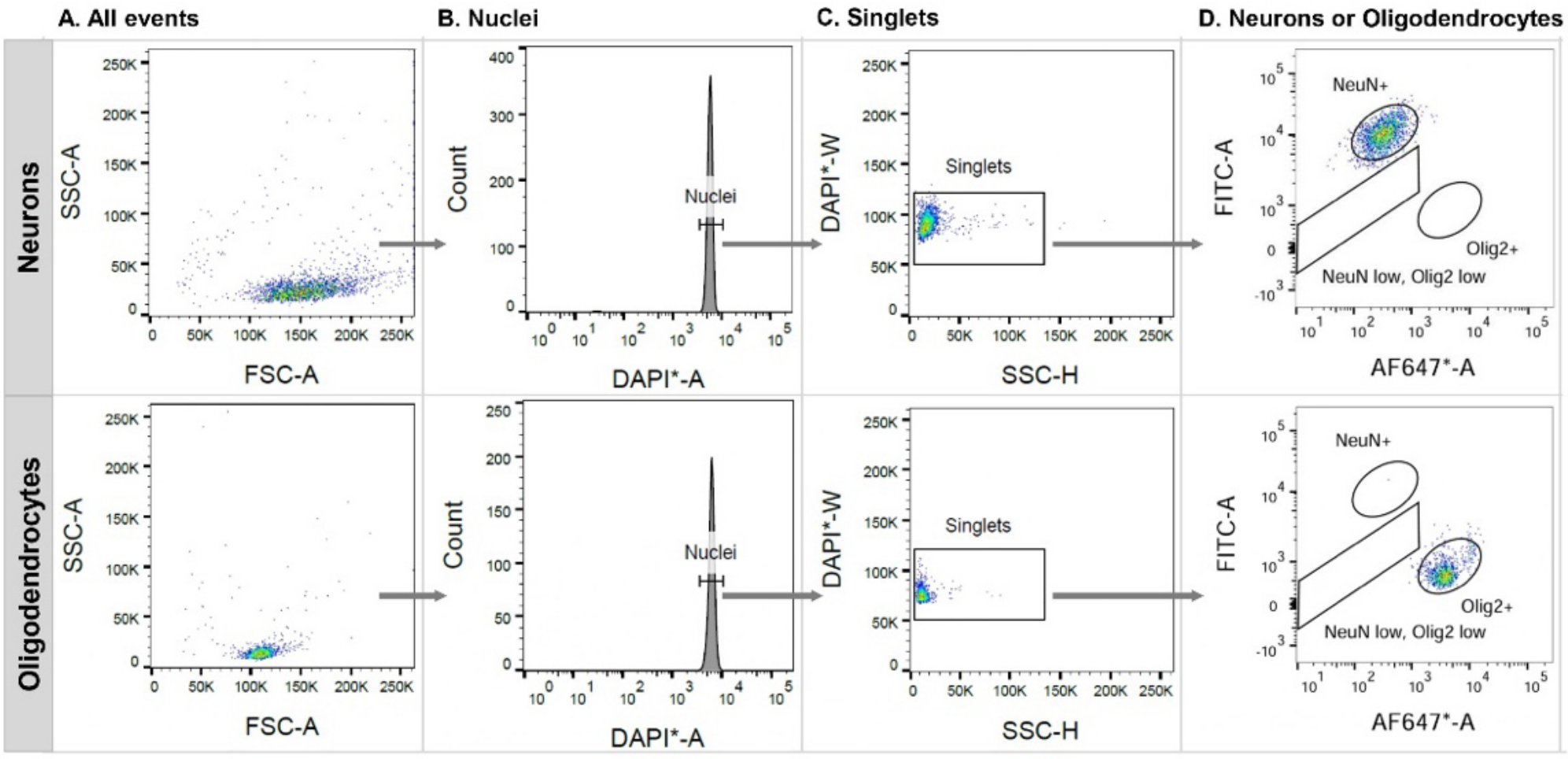

Figure S9. Purity assessment of FACS-sorted neuronal and oligodendroglial nuclei from mouse cortex. Representative flow cytometry plots showing the gating strategy and post-sort purity analysis of nuclei isolated from mouse cerebral cortex and stained with DAPI and antibodies against NeuN (FITC), Olig2 (AF647), and SOX9 (PE). **A**. All events, including nuclei and debris, were visualized on a side scatter area (SSC-A) versus forward scatter area (FSC-A) dot plot. **B**. DAPI-positive nuclei were identified and debris excluded using a DAPI area (DAPI-A) histogram. **C**. Singlet nuclei were selected to exclude aggregates using a DAPI width (DAPI-W) versus SSC height (SSC-H) dot plot. **D**. Singlet neuronal- and oligodendroglial-derived nuclei were identified on an anti-NeuN FITC-A versus anti-Olig2 AF647-A dot plot. Astrocyte-derived nuclei were defined as NeuN low/Olig2 low/SOX9^{+} (not shown). Upper panels show re-analysis of sorted neuronal nuclei, which localize predominantly within the NeuN^{+} gate. Lower panels show re-analysis of sorted oligodendroglial nuclei, which localize predominantly within the Olig2^{+} gate. Post-sort re-analysis of astrocytic (SOX9^{+}) nuclei was not performed due to limited yield after sorting. Gates were defined using appropriate single-stained and unstained controls. Plots are representative of independent experiments.

Droplet digital PCR

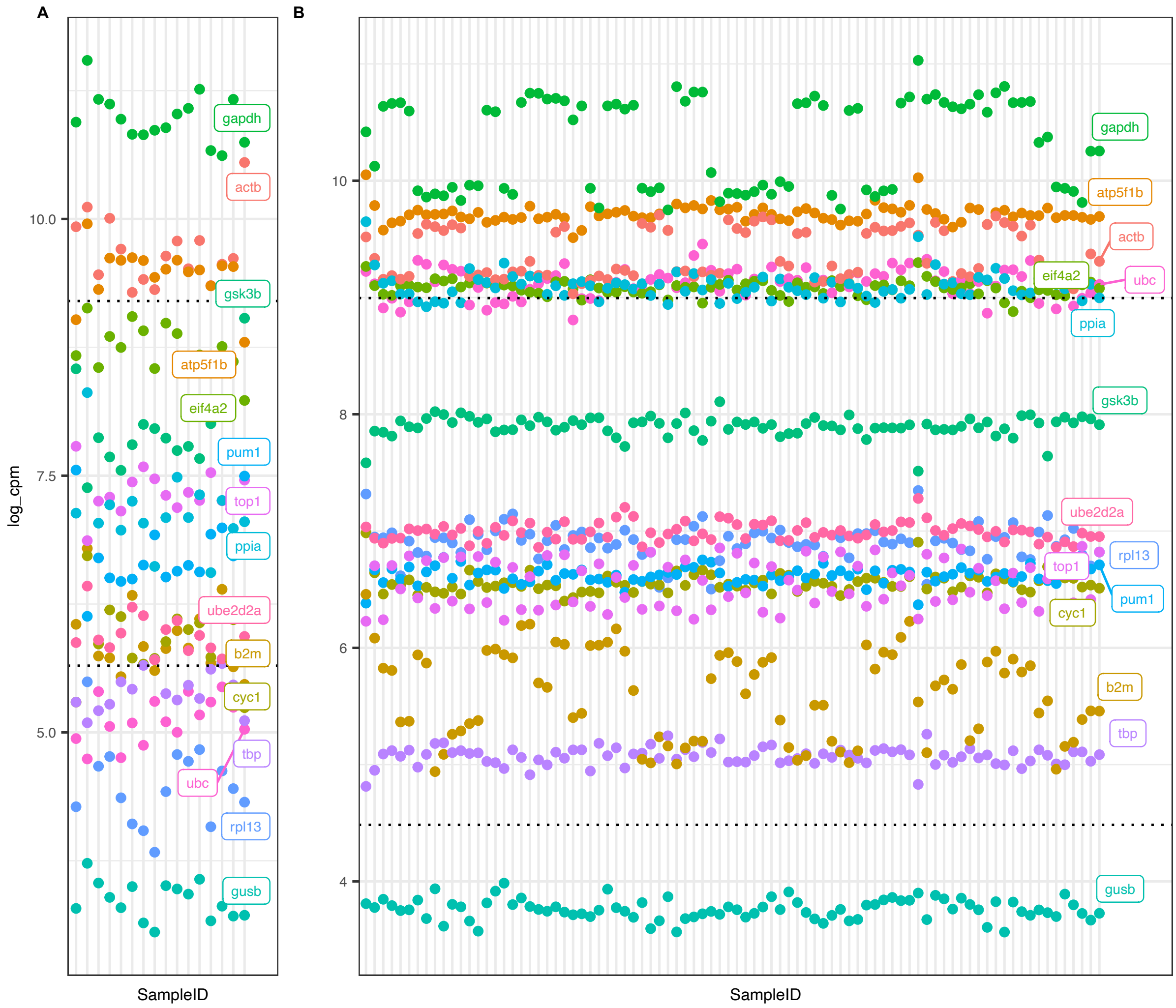

Figure S10: Reference gene stability
Gene expression of reference gene candidates from the RNAseq datasets: **A.** Mouse cortex (n=16) from wildtype (not published), and **B.** Mouse cortex (n= 86) of wildtype mouse with or without sleep deprivation from Diessler et al[1]. Dotted lines indicate mean expression of the *Ank3* gene (upper line) and the Astrocyte marker *Sox9* (lower line). Refer to table S10 for standard deviation (sd) and coefficient of variation (CV) for each gene.

A

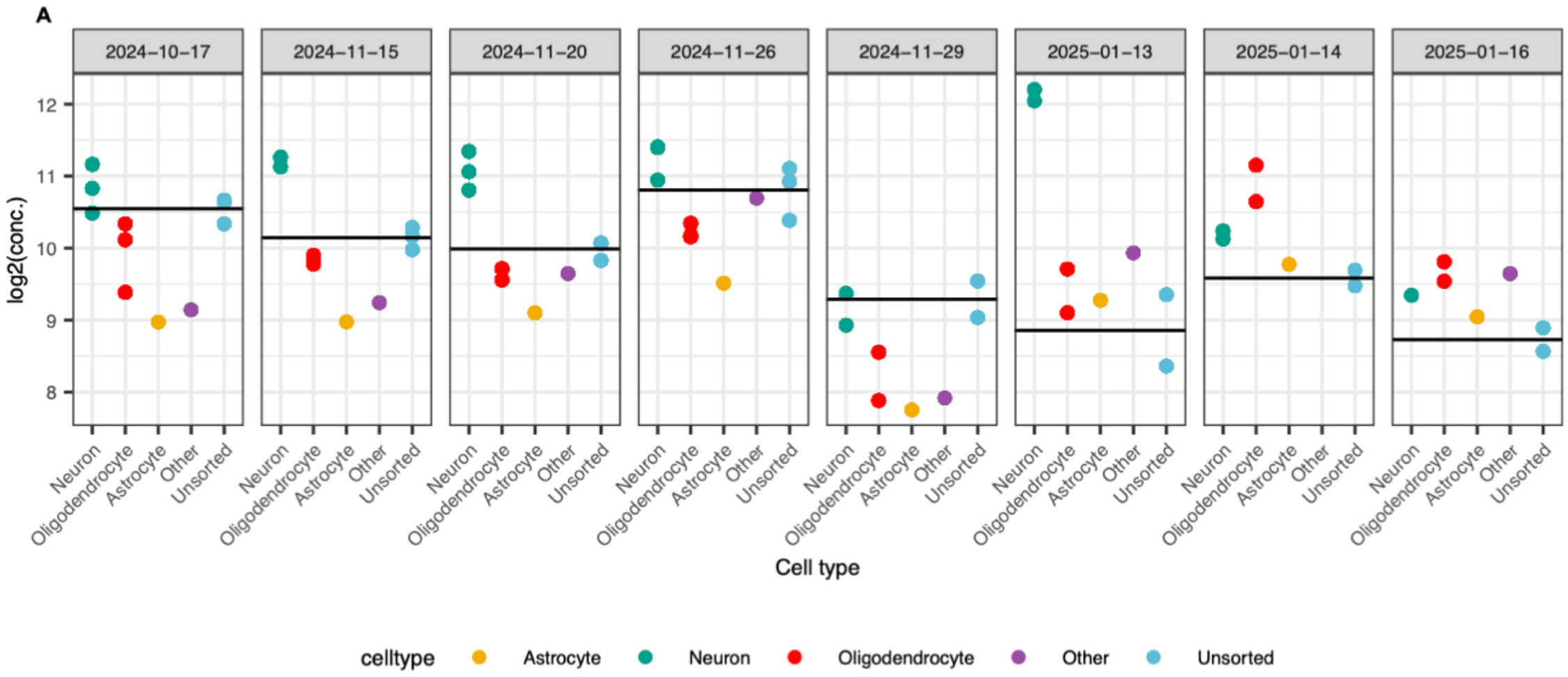

B

celltype	Top1_factor_log2	Top1_factor
Neuron	0.654	1.574
Oligodendrocyte	-0.399	0.758
Astrocyte	-0.958	0.515
Unknown	-1.013	0.495
Unsorted	0.000	1.000

C

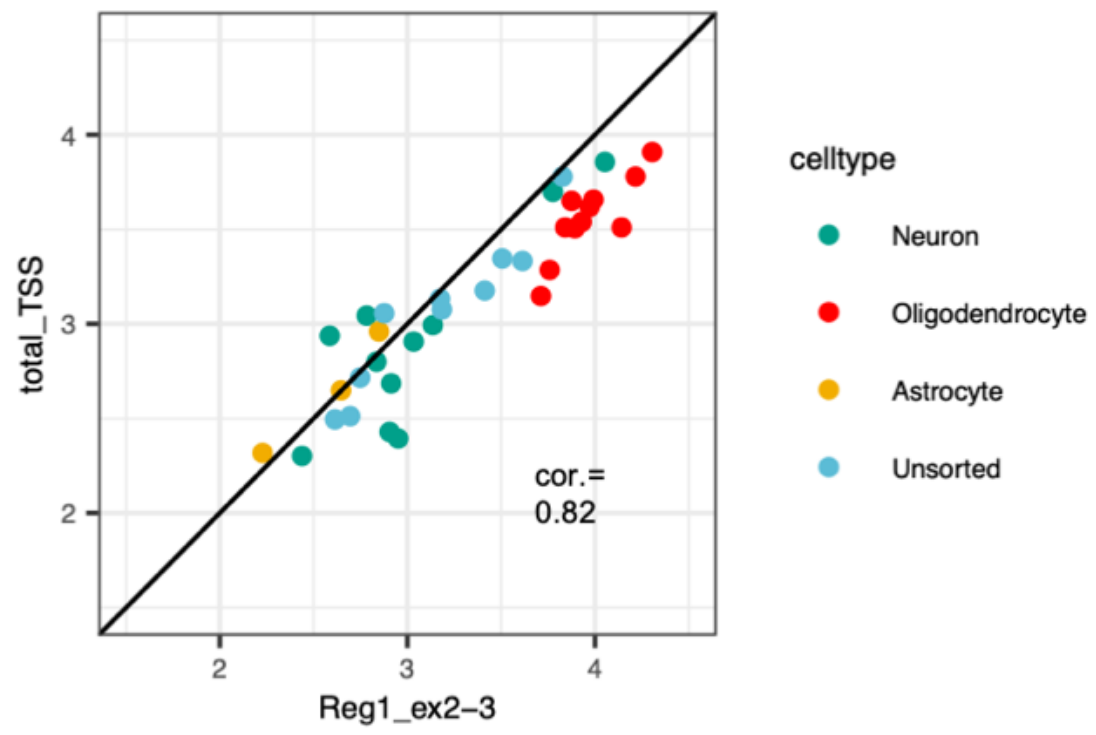

Figure S11: Total *Ank3* expression and Top1 adjustment factors
A. Top1 expression in each cell type for each nuclei sorting experiment. Horizontal line indicates mean expression of unsorted nuclei (DAPI). Top1 adjustment factor for each cell type is median quotient of mean expression to Unsorted mean expression. **B.** The adjustment factors used for the reference gene Top1, to compensate for consistent differences in its expression by cell type. **C.** The adjusted expression of the combined transcription start sites (TSS1 + TSS2) plotted against the constitutive exon measured by Reg1_ex2-3, ENSMUSE00001258730. The diagonal line indicates equal expression.

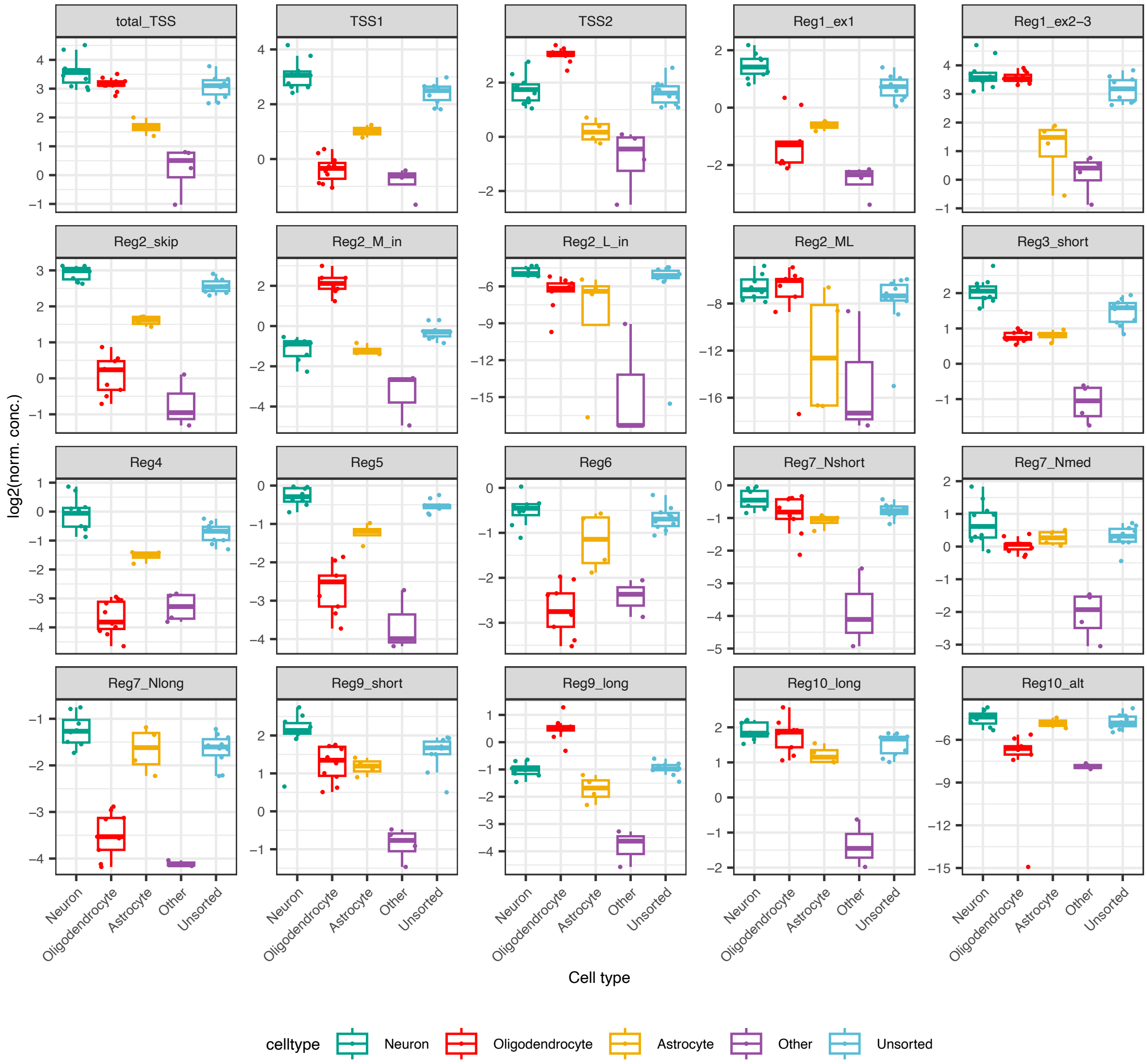

Figure S12: *Ank3* isoform expression between cell types, all measurements
Comparison of the expression of several *Ank3* isoforms in different cell type populations. Expression in log2 counts per ng RNA, normalised to the adjusted Top1 reference gene.
